# Reliability-Weighted Integration of Audiovisual Signals Can Be Modulated by Top-down Attention

**DOI:** 10.1523/ENEURO.0315-17.2018

**Published:** 2018-03-08

**Authors:** Tim Rohe, Uta Noppeney

**Affiliations:** 1 Max Planck Institute for Biological Cybernetics, Tübingen, 72076, Germany; 2 Department of Psychiatry and Psychotherapy, University of Tübingen, Tübingen, Germany; 3 Computational Neuroscience and Cognitive Robotics Centre, University of Birmingham, Birmingham, United Kingdom

**Keywords:** Intraparietal sulcus, multisensory integration, optimal cue integration

## Abstract

Behaviorally, it is well established that human observers integrate signals near-optimally weighted in proportion to their reliabilities as predicted by maximum likelihood estimation. Yet, despite abundant behavioral evidence, it is unclear how the human brain accomplishes this feat. In a spatial ventriloquist paradigm, participants were presented with auditory, visual, and audiovisual signals and reported the location of the auditory or the visual signal. Combining psychophysics, multivariate functional MRI (fMRI) decoding, and models of maximum likelihood estimation (MLE), we characterized the computational operations underlying audiovisual integration at distinct cortical levels. We estimated observers’ behavioral weights by fitting psychometric functions to participants’ localization responses. Likewise, we estimated the neural weights by fitting neurometric functions to spatial locations decoded from regional fMRI activation patterns. Our results demonstrate that low-level auditory and visual areas encode predominantly the spatial location of the signal component of a region’s preferred auditory (or visual) modality. By contrast, intraparietal sulcus forms spatial representations by integrating auditory and visual signals weighted by their reliabilities. Critically, the neural and behavioral weights and the variance of the spatial representations depended not only on the sensory reliabilities as predicted by the MLE model but also on participants’ modality-specific attention and report (i.e., visual vs. auditory). These results suggest that audiovisual integration is not exclusively determined by bottom-up sensory reliabilities. Instead, modality-specific attention and report can flexibly modulate how intraparietal sulcus integrates sensory signals into spatial representations to guide behavioral responses (e.g., localization and orienting).

## Significance Statement

To obtain an accurate representation of the environment, the brain should integrate noisy sensory signals by weighting them in proportion to their relative reliabilities. This strategy is optimal by providing the most reliable (i.e., least variable) percept. The extent to which the brain top-down controls the sensory weights in the integration process remains controversial. The current study shows that the parietal cortex weighs audiovisual signals by their reliabilities. Yet the sensory weights and the variance of the multisensory representations were also influenced by modality-specific attention and report. These results suggest that audiovisual integration can be flexibly modulated by top-down control.

## Introduction

In our natural environment our senses are continuously exposed to noisy sensory signals that provide uncertain information about the world. To construct a veridical representation of the environment, the brain is challenged to integrate sensory signals if they pertain to common events. Numerous psychophysics studies have demonstrated that human observers combine signals within and across the senses by weighting them in proportion to their reliabilities, with greater weights assigned to the more reliable signal (i.e., the inverse of a signal’s variance; Jacobs, 1999; Ernst and Banks, 2002; Knill and Saunders, 2003; Alais and Burr, 2004). If two signals provide redundant information about the same event (i.e., common-source assumption), this reliability-weighted multisensory integration provides the most precise, i.e., statistically optimal, perceptual estimate [i.e., maximum likelihood estimate (MLE)], leading to better performance on a range of tasks such as depth (Ban et al., 2012), shape (Ernst and Banks, 2002), motion (Fetsch et al., 2012), or spatial (Alais and Burr, 2004) discrimination. However, reliability-weighted integration is statistically optimal only for the special case where a single cause elicited the signals, i.e., the common-source assumptions are met. In our natural environment, two signals can arise from either common or separate sources, leading to some uncertainty about the causal structure underlying the sensory signals. Mandatory integration of sensory signals would in many instances effectively misattribute information (Roach et al., 2006). In a more natural context, the observer has to infer the causal structure from sensory correspondences such as spatial colocation (Wallace et al., 2004) or temporal correlation (Parise and Ernst, 2016). The observer should then integrate signals in case of a common cause but segregate them in case of independent causes (Körding et al., 2007). In other words, reliability-weighted integration is no longer statistically optimal in more general situations where the causal structure of the sensory signals is unknown or the assumption of a common source is violated.

Despite abundant behavioral evidence for near-optimal reliability-weighted integration under experimental conditions which foster the assumption of a common signal cause, the underlying neural mechanisms remain unexplored in the human brain for multisensory signals. For cue combination within a single sensory modality, higher-order visual regions have recently been implicated in reliability-weighted integration of visual-depth cues (Ban et al., 2012). Only recently, elegant neurophysiological studies in nonhuman primates have started to characterize the neural mechanisms of visual-vestibular integration for heading discrimination. They demonstrated that single neurons (Morgan et al., 2008) and neuronal populations (Fetsch et al., 2012) in the dorsal medial superior temporal area (dMST) integrated visual and vestibular motion near-optimally weighted by their reliabilities. Moreover, the neural weights derived from neural population responses in dMST corresponded closely to the weights governing monkeys’ behavioral choices.

Over the past decade, accumulating evidence has shown that multisensory integration is not deferred until later processing stages in higher-order association cortices (Beauchamp et al., 2004; Sadaghiani et al., 2009), but starts already at the primary cortical level (Foxe et al., 2000; Ghazanfar and Schroeder, 2006; Kayser et al., 2007; Lakatos et al., 2007; Lewis and Noppeney, 2010; Werner and Noppeney, 2010; Lee and Noppeney, 2014). Previous functional imaging research indicated in a qualitative fashion that sensory reliability modulates regional blood oxygenation level–dependent (BOLD) responses (Helbig et al., 2012), functional connection strengths (Nath and Beauchamp, 2011), or activation patterns (Ban et al., 2012; Rohe and Noppeney, 2016). For instance, during speech recognition, the superior temporal sulcus coupled more strongly with the auditory cortex when auditory reliability was high but with visual cortex when visual reliability was high (Nath and Beauchamp, 2011). Likewise, using functional MRI (fMRI) multivariate pattern decoding, a recent study showed that parietal cortices integrated spatial signals depending on their spatial disparity and sensory reliability (Rohe and Noppeney, 2016). However, to our knowledge, no previous study has evaluated whether multisensory integration in the human brain follows the quantitative predictions of the MLE model.

Computational models of probabilistic population coding (Ma et al., 2006) suggest that reliability-weighted integration may be obtained by averaging the inputs with fixed weights from upstream populations of neurons that encode the reliability of the sensory input in terms of the sensory gain. By contrast, the recently proposed normalization model of multisensory integration (Ohshiro et al., 2011, 2017) suggests that normalization over a pool of neurons as a canonical computational operation can implement multisensory integration with weights that flexibly adjust to the reliability of the sensory inputs. Critically, in both models, reliability-weighted integration depends on a region having access to inputs from upstream regions that are responsive to auditory and visual inputs. Although accumulating evidence suggests that multisensory integration starts already at the primary cortical level (Foxe et al., 2000; Bonath et al., 2007, 2014; Kayser et al., 2007; Lakatos et al., 2007; Lewis and Noppeney, 2010; Werner and Noppeney, 2010; Lee and Noppeney, 2014), the fraction of multisensory neurons that are influenced by inputs from multiple sensory modalities increases across the cortical hierarchy (Bizley et al., 2007; Dahl et al., 2009). Thus, even if low-level sensory areas are susceptible to limited influence from other sensory modalities, this activity may be less informative (i.e., more unreliable) than that of the preferred sensory modality. As a result, reliability-weighted integration via normalization may be more prominent in higher-order association cortices than in low-level sensory areas.

Besides the assumption of a common signal cause, a second assumption of the classic MLE model is that the sensory weights and the variance reduction obtained from multisensory integration depend solely on the bottom-up reliabilities of the sensory inputs irrespective of cognitive influences (i.e., the unisensory reliabilities are not influenced by observers’ attentional focus, e.g., selective vs. divided attention). In line with this conjecture, initial psychophysical studies suggested that the sensory weights are immune to attentional influences (Helbig and Ernst, 2008). Yet, more recent psychophysical studies have demonstrated that the sensory weights are modulated by attentional top-down effects (Vercillo and Gori, 2015). Moreover, electroencephalography (EEG) and fMRI studies revealed profound attentional effects on the neural processes underlying multisensory integration (Talsma et al., 2010; Donohue et al., 2011). The controversial results raise the question of whether the task-relevance of sensory signals influences reliability-weighted integration at the neural level even if the signals’ small disparity suggests a common cause.

The present study combined psychophysics and fMRI multivariate decoding to characterize the neural processes underlying multisensory integration in a quantitative fashion and to investigate potential top-down effects of modality-specific report and associated attentional effects. We presented participants with auditory, visual, and audiovisual signals that were spatially congruent or in a small spatial conflict. On each trial, participants were presented with an auditory and a visual spatial signal from four possible horizontal locations. They located either the visual or the auditory signal by pushing one of four response buttons that corresponded to the four locations. To compute psychometric functions, participants’ responses were binarized into left-versus-right responses. To assess top-down effects of modality-specific report on the behavioral and neural weights, we manipulated whether participants reported the auditory or visual locations. In a model-based analysis, we first investigated whether the sensory weights and variances obtained from psychometric and neurometric functions were in line with the predictions of the MLE model. In a model-free analysis, we next examined whether the sensory weights and variances were influenced by visual signal reliability and/or report of the auditory (or visual) modality.

## Materials and Methods

### Participants

After giving written informed consent, six healthy volunteers (two females, mean age 28.8 yr, range 22–36 yr) participated in the fMRI study. All participants had normal or corrected-to normal vision and reported normal hearing. One participant was excluded due to excessive head motion (4.21/3.52 STD above the mean of the translational/rotational volume-wise head motion based on the included 5 participants). The study was approved by the human research review committee of the University of Tübingen. A subset of the data (i.e., the audiovisual conditions) has been reported in Rohe and Noppeney (2015a, 2016).

### Stimuli

The visual stimulus was a cloud of 20 white dots (diameter: 0.43° visual angle) sampled from a bivariate Gaussian with a vertical standard deviation of 2.5° and a horizontal standard deviation of 2° or 14° (high and low visual reliability). The visual stimulus was presented on a black background (i.e., 100% contrast). The auditory stimulus was a burst of white noise with a 5-ms on/off ramp. To create a virtual auditory spatial signal, the noise was convolved with spatially specific head-related transfer functions (HRTFs). The HRTFs were pseudo-individualized by matching participants’ head width, height, depth, and circumference to the anthropometry of participants in the CIPIC database (Algazi et al., 2001) and were interpolated to the desired location of the auditory signal.

### Experimental design and procedure

In the unisensory conditions, participants were presented with either auditory or visual signals of low or high reliability. The signals were sampled from four possible locations along the azimuth (–10°, –3.3°, 3.3°, or 10°). This yielded 4 auditory conditions (i.e., 4 auditory locations) and 4 visual locations × 2 visual reliability levels (high vs. low) = 8 visual conditions. On each trial, participants located either the visual or the auditory signal.

**Figure 1.**
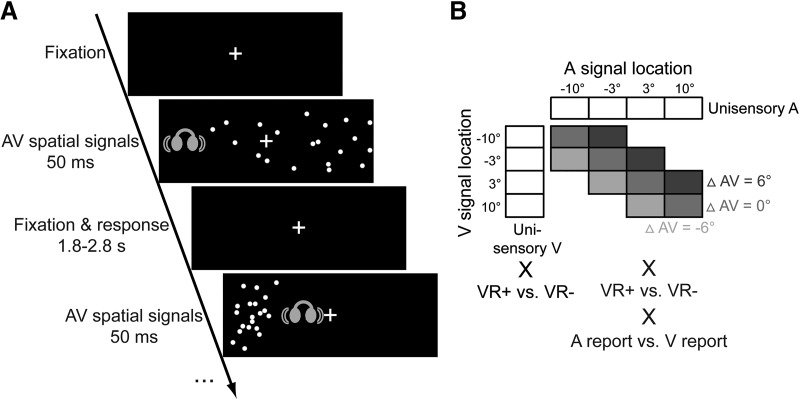
Example trial and experimental design. ***A***, Participants were presented with unisensory auditory, unisensory visual and synchronous audiovisual signals originating from four possible locations along the azimuth. The visual signal was a cloud of white dots. The auditory signal was a brief burst of white noise presented via headphones. Participants localized either the auditory or the visual signal (note that for illustrational purposes the visual angles of the cloud have been scaled in a non-uniform fashion in this scheme). ***B***, In the audiovisual conditions, the experimental design manipulated (1) the location of the visual (V) signal (−10°, −3.3°, 3.3°, 10°; 2) the location of the auditory (A) signal (−10°, −3.3°, 3.3°, 10°), (3) the reliability of the visual signal (low versus high standard deviation of the visual cloud; VR+ vs. VR-), and (4) modality-specific report (auditory versus visual). Only congruent (△AV = 0°; △AV = A - V) and slightly disparate conditions (△AV = ±6°) were used in this study. In unisensory conditions, the experimental design manipulated the location of the auditory signal in auditory conditions and the locations of the visual signals as well as visual reliability in visual conditions.

In the audiovisual conditions, participants were presented with synchronous auditory and visual signals of high or low visual reliabilities (Fig. 1*A*). They attended and reported the location of either the visual or auditory signal component. The locations of the auditory and visual signal components were sampled independently from four possible locations. This yielded 4 auditory locations × 4 visual locations = 16 audiovisual location combinations that varied in their audiovisual spatial disparities. In the current study, we focused selectively on the audiovisually congruent (A-V = ΔAV = 0°) and slightly conflicting (ΔAV = 6° and = –6°) conditions. These small, so-called nonnoticeable, spatial conflicts have previously been introduced to test the predictions of the maximum likelihood estimation (MLE) model (e.g., Battaglia et al., 2003; Alais and Burr, 2004), as they are assumed to ensure that observers fuse sensory signals into one unified percept. Note that results of the audiovisual conditions with larger disparity (ΔAV > 6°) have been reported in Rohe and Noppeney (2015a, 2016).

**Figure 2.**
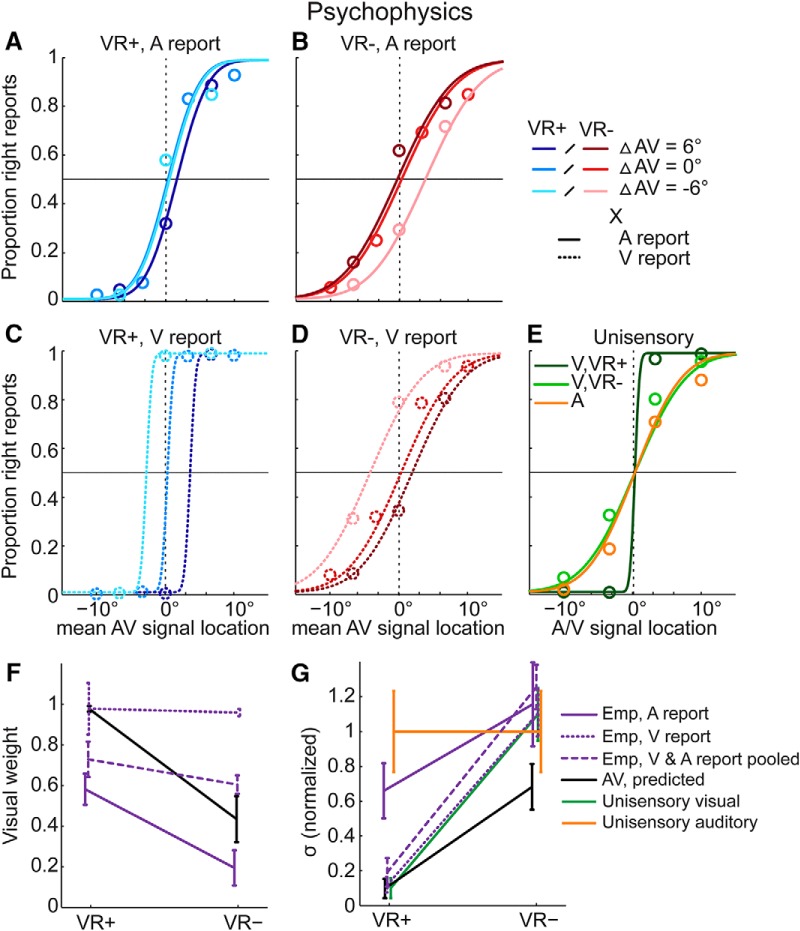
Psychophysics results: psychometric functions, visual weights and audiovisual variances. In audiovisual (AV) conditions, psychometric functions were fitted to the fraction of “right” location responses plotted as a function of the mean AV location. Data were fitted separately for audiovisual spatially congruent (△AV = 0°) and slightly conflicting conditions (△AV = ±6° with △AV = A - V). The empirical visual weight is computed from PSE locations of the audiovisual spatially conflicting psychometric functions (see equation 2). If the visual weight is >0.5, the PSE for △AV = -6° is left of the PSE for △AV = 6°. If the visual weight is smaller than 0.5, the PSE for △AV = -6° is right of the PSE for △AV = 6°. If the visual weight is equal to 0.5, the PSEs for △AV = -6° and △AV = 6° are identical. ***A–D***, Psychometric functions for audiovisual spatially congruent and conflicting trials are plotted separately for the four conditions in our 2 (visual reliability: high, VR+ vs. low, VR-) x 2 (modality-specific report: auditory versus visual) factorial design. ***E***, In unisensory conditions, psychometric functions were fitted to the fraction of “right” location responses plotted as a function of the signal location from unisensory auditory (A) and visual conditions of high (V, VR+) and low (V, VR-) visual reliability. ***F***, Visual weights (mean ± SEM across participants): MLE predicted and empirical weights for the four conditions in our 2 (visual reliability: high, VR+ vs. low, VR-) x 2 (modality-specific report: auditory versus visual) factorial design. To facilitate the comparison with the MLE predictions that do not depend on modality-specific report, the visual weights are also plotted after pooling the data across both report conditions and re-fitting the neurometric functions. ***G***, Standard deviations (σ, mean ± SEM across participants): Unisensory and audiovisual MLE-predicted and empirical standard deviations of the perceived spatial locations for the same combination of conditions as in ***F***. For illustrational purposes, standard deviations were normalized by the auditory standard deviation (original auditory standard deviatio*n* = 39 ± 1.25; mean ± SEM).

**Figure 3.**
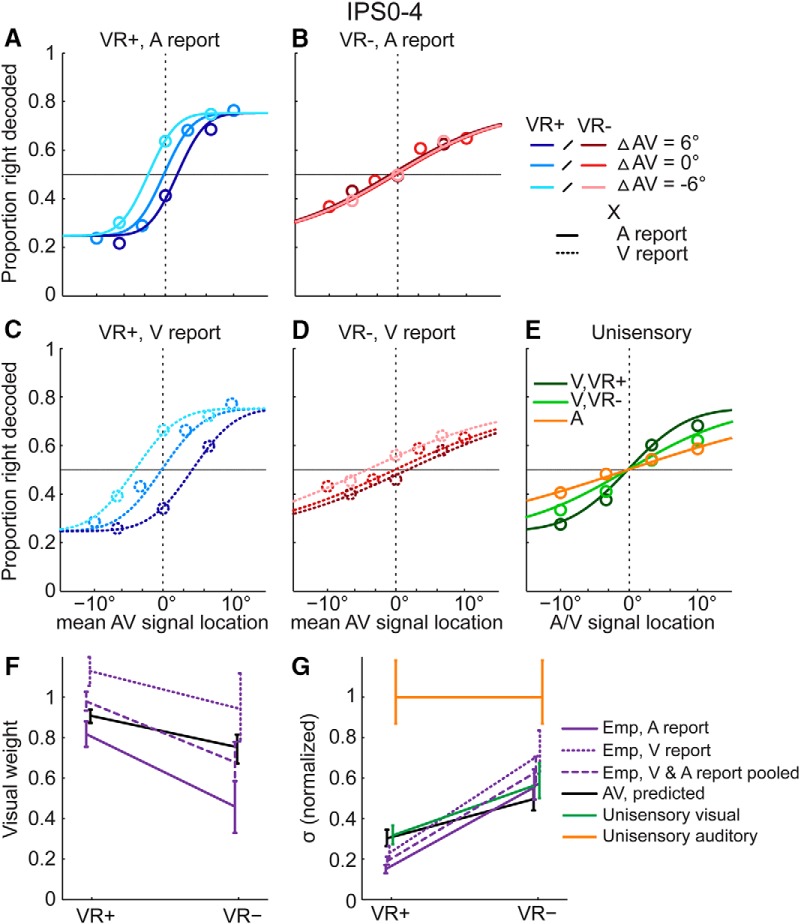
fMRI results in the intraparietal sulcus: neurometric functions, visual weights and audiovisual variances. In intraparietal sulcus (IPS0–4), neurometric functions were fitted to the fraction of decoded “right” location responses plotted as a function of the mean audiovisual (AV) location (see figure 2 legend for additional information). ***A–D***, Neurometric functions are plotted separately for the four conditions in our 2 (visual reliability: high, VR+ vs. low, VR-) x 2 (modality-specific report: auditory versus visual) factorial design. ***E***, In unisensory conditions, psychometric functions were fitted to the fraction of “right” location responses plotted as a function of the signal location from unisensory auditory (A) and visual conditions of high (V, VR+) and low (V, VR-) visual reliability. ***F***, Visual weights (mean and 68% bootstrapped confidence interval): MLE-predicted and empirical visual weights for 2 (visual reliability: high, VR+ vs. low, VR-) x 2 (modality-specific report: auditory versus visual) AV conditions. To facilitate the comparison with the MLE predictions that do not depend on modality-specific report, the visual weights are also plotted after pooling the data across both report conditions and re-fitting the neurometric functions. ***G***, Standard deviations (σ, mean and 68% bootstrapped confidence interval): Unisensory and audiovisual MLE predicted and empirical standard deviations for the same combination of conditions as in ***F***. For illustrational purposes, standard deviations were normalized by the auditory standard deviation (original auditory standard deviation = 21.54). For extended analyses controlling motor responses and global interhemispheric activation differences in IPS0–4, see Fig. 3-1 and 3-2.

**Figure 4.**
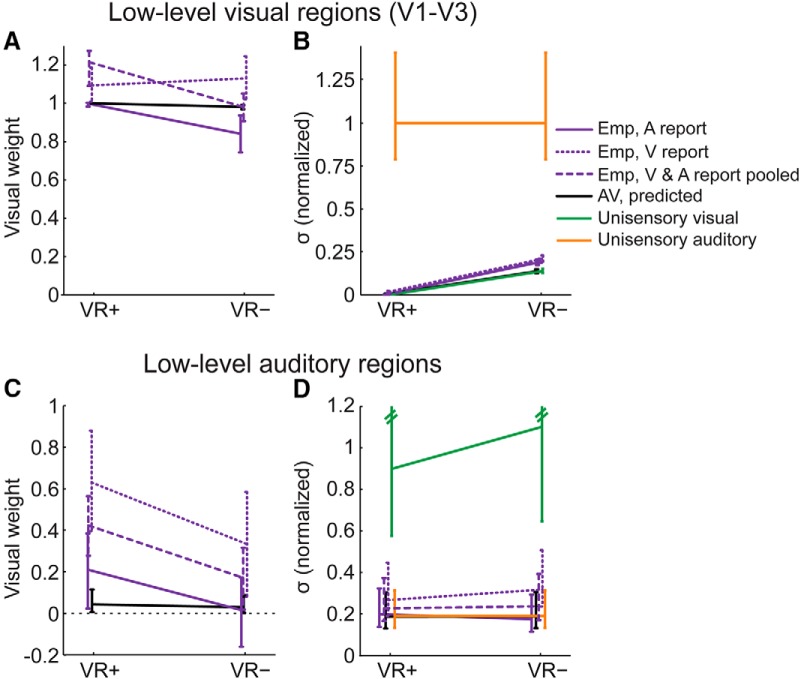
fMRI results in low-level visual and auditory regions: Visual weights and audiovisual variances. ***A***, Visual weights (mean and 68% bootstrapped confidence interval): MLE predicted and empirical visual weights for 2 (visual reliability: high, VR+ vs. low, VR-) x 2 (modality-specific report: auditory versus visual) audiovisual conditions in low-level visual regions (V1-3). To facilitate the comparison with the MLE predictions that do not depend on modality-specific report, the visual weights are also plotted after pooling the data across both report conditions and re-fitting the neurometric functions. ***B***, Standard deviations (σ, mean and 68% bootstrapped confidence interval): Unisensory and audiovisual MLE predicted and empirical standard deviations for the same combination of conditions as in ***A***. For illustrational purposes, standard deviations were normalized by the auditory standard deviation (original auditory standard deviation = 61.68). ***C***, Visual weights (mean and 68% bootstrapped confidence interval): MLE predicted and empirical visual weights in low-level auditory regions (hA) as shown in ***A***. ***D***, Standard deviations (σ, mean and 68% bootstrapped confidence interval): Unisensory and audiovisual MLE predicted and empirical standard deviations of spatial representations in low-level auditory regions (hA) as shown in ***B***; note that the upper confidence interval for the visual variance is truncated for illustrational purposes. For illustrational purposes, standard deviations were normalized by a combined visual standard deviation for low and high visual reliability (original visual standard deviation = 38.75, averaged across levels of visual reliability). For extended analyses controlling for global interhemispheric activation differences in low-level visual and auditory regions, see Fig. 4-1.

In total, this MLE study included 52 conditions (Fig. 1*B*)—4 unisensory auditory conditions, 4 unisensory visual conditions of high visual reliability, 4 unisensory visual conditions of low visual reliability—and 40 audiovisual conditions—(4 audiovisually congruent + 6 audiovisually incongruent conditions with a small spatial disparity) × 2 visual reliability levels (high vs. low) × 2 modality-specific reports (visual vs. auditory). For the model-free analysis, we obtained variances and sensory weights by fitting psychometric and neurometric functions separately to the perceived and decoded spatial locations (i.e., percentage perceived right as a function of spatial location) separately for the four conditions in a 2 (visual reliability: high vs. low) × 2 (modality-specific report: auditory vs. visual) factorial design.

On each trial, audiovisual signals were presented for 50 ms duration with a variable interstimulus fixation interval of 1.75–2.75 s (Fig. 1*A*). Participants reported their auditory perceived location in the unisensory auditory and the audiovisual sessions with auditory report. They reported their visual perceived location in the unisensory visual and the audiovisual sessions with visual report. Participants indicated their perceived location by pushing one of four buttons that spatially corresponded to the four signal locations (–10°, –3.3°, 3.3°, or 10° along the azimuth) using their right hand. To compute psychometric functions, participants’ responses were binarized into left-versus-right responses for all analyses. Throughout the experiment, participants fixated on a central cross (1.6° diameter).

Unisensory and audiovisual stimuli were presented in separate sessions. Subjects participated in 3–4 unisensory auditory, 3–4 unisensory visual, and 20 audiovisual sessions (10 auditory and 10 visual report, except one participant who performed 9 auditory and 11 visual report sessions). In the respective sessions, we presented the 4 unisensory auditory conditions in 88 trials each, the 8 unisensory visual conditions in 44 trials each, and the 32 audiovisual conditions (4 visual stimulus locations × 4 auditory stimulus locations × 2 visual reliability levels) in 11 trials each. Further, 5.9% null events (i.e., pseudo-events without a stimulation) were interspersed in the sequence of 352 stimuli per session to estimate stimulus-evoked responses relative to the fixation baseline. To maximize design efficiency, trial types were presented in a pseudorandomized order. We manipulated the modality-specific report (visual vs. auditory) over sessions in a counterbalanced order within each participant and presented unisensory and audiovisual runs in a counterbalanced order across participants.

### Experimental setup

Audiovisual signals were presented using Psychtoolbox 3.09 (www.psychtoolbox.org; Brainard, 1997; Kleiner et al., 2007) running under Matlab R2010a (MathWorks). Auditory stimuli were presented at ∼75 dB SPL using MR-compatible headphones (MR Confon). Visual stimuli were back-projected onto a Plexiglas screen using an LCoS projector (JVC DLA-SX21). Participants viewed the screen through an extra-wide mirror mounted on the MR head coil, resulting in a horizontal visual field of ∼76° at a viewing distance of 26 cm. Participants indicated their response using an MR-compatible custom-built button device. Participants’ eye movements and fixation were monitored by recording participants’ pupil location using an MR-compatible custom-built infrared camera (sampling rate 50 Hz) mounted in front of the participants’ right eye and iView software 2.2.4 (SensoMotoric Instruments).

### Key predictions of the MLE model

The majority of multisensory research today has focused on the so-called forced fusion case, in which observers *a priori* assume that two signals come from a common source and should hence be integrated. These forced fusion criteria are generally assumed to be met when observers are instructed to locate a single source that emits audiovisual signals (i.e., bisensory attention) and the two signals are presented without any conflict or with a small cue conflict such as a spatial disparity of 6° visual angle as employed in our experiment (e.g., Alais and Burr, 2004). Under these classic forced fusion assumptions, the MLE model makes two key quantitative predictions for participants’ spatial estimates that are formed by integrating auditory and visual signals. The first prediction pertains to the sensory weights applied during the integration process, and the second to the variance of the integrated perceived signal location.

First, the most reliable unbiased estimate of an object’s location (S^AV) is obtained by combining the auditory (S^A) and visual (S^V) perceived locations in proportion to their relative reliabilities (*r_A_*, *r_V_*; i.e., the inverse of the variance, *r* = 1/σ^2^):

(1) S^AV=wAS^A+wVS^V,

with

$$w_{A}=\frac{r_{A}}{r_{A}+r_{V}}=\frac{\frac{1}{{\sigma_{A}}^{2}}}{\frac{1}{{\sigma_{A}}^{2}}+\frac{1}{{\sigma_{V}}^{2}}}$$

and

$$w_{V}=\frac{r_{V}}{r_{A}+r_{V}}=\frac{\frac{1}{{\sigma_{V}}^{2}}}{\frac{1}{{\sigma_{A}}^{2}}+\frac{1}{{\sigma_{V}}^{2}}}$$

The variances obtained from the cumulative Gaussians that were fitted to the unisensory visual and auditory conditions were used to determine the optimal weights that participants should apply to the visual and auditory signals in the audiovisual conditions as predicted by the MLE model [Eq. (1)]. The empirical weights were computed from the point of subjective equality (PSE) of the psychometric functions of the audiovisual conditions where a small audiovisual spatial disparity of 6° was introduced according to the following equation (Helbig and Ernst, 2008; Fetsch et al., 2012):

(2) $$w_{V,emp}=\frac{{PSE}_{\Delta AV=+6{}^{\circ}}-{PSE}_{\Delta AV=-6{}^{\circ}}}{2\Delta AV}+\frac{1}{2}$$

Note that the equation assumes that the psychometric functions plot percentage perceived right as a function of the average of the true auditory and visual locations (Figs. 2 and 3).

Second, multisensory integration reduces the variance of the audiovisual estimate (σ*_AV_*^2^), in particular for congruent audiovisual trials, compared with the unisensory variances (σ*_A_*^2^, σ*_V_*^2^):

(3) $${\sigma_{AV}}^{2}=\frac{{\sigma_{A}}^{2}{\sigma_{V}}^{2}}{{\sigma_{A}}^{2}+{\sigma_{V}}^{2}}$$

To generate MLE predictions for the audiovisual variance, the unisensory variances were obtained from the psychometric functions (i.e., cumulative Gaussians) for the auditory and visual signals. The empirical variance of the combined audiovisual estimate was obtained from the psychometric function for the audiovisual conditions.

### Behavioral data

Participants’ spatial location responses (four buttons) were categorized as “left” (i.e., −10° or −3.3°) or “right” (i.e., 3.3° or 10°) responses. For the unisensory auditory and visual conditions, we plotted the fraction of “right” responses as a function of the unisensory signal location (Fig. 2*E*). For the audiovisual spatially congruent and conflicting conditions, we plotted the fraction of “right” responses as a function of the mean signal location of the true auditory and true visual signal locations (separately for the 4 conditions in our 2 (auditory vs. visual report) × 2 (high vs. low visual reliability) factorial design; Fig. 2*A–D*).

For the behavioral analysis, we fitted cumulative Gaussian functions individually to the data of each participant (again separately for the 4 conditions in our 2 (auditory vs. visual report) × 2 (high vs. low visual reliability) factorial design using maximum likelihood estimation methods as implemented in Palamedes toolbox 1.5.0 (Prins and Kingdom, 2009). To enable reliable parameter estimation for each participant, we employed the following constraints: (1) The Gaussians’ means [i.e., point of subjective equality (PSE)] were constrained to be equal across unisensory and audiovisual congruent conditions (i.e., identical spatial biases were assumed across unisensory and audiovisual congruent conditions). (2) The Gaussians’ variances (i.e., perceptual thresholds or slopes of the psychometric functions) were constrained to be equal for the congruent and the two conflicting conditions within each combination of visual reliability and modality-specific report. Note that this is based on the fundamental forced-fusion assumption implicitly adopted in previous research (Ernst and Banks, 2002; Alais and Burr, 2004) whereby the conditions with small nonnoticeable cue conflict are considered to be equivalent to congruent conditions. (3) Guess and lapse rate parameters were set to be equal (i.e., guess = lapse rate) and constrained to be equal across all conditions. In other words, we assumed that observers possibly made false responses (e.g., a “right” response for a signal at –10°) for nonspecific reasons such as blinking, inattention, etc., with equal probability in their outer left and right hemifields. Based on those constraints, we fitted 17 parameters to the 52 data points individually for each participant. More specifically, we fitted one PSE parameter commonly for the unisensory visual, auditory, and audiovisual congruent conditions, one PSE parameter each for the 8 conflict conditions (i.e., 2 visual reliability × 2 modality-specific report × 2 spatial conflict, Δ*AV* = –6° or 6°; i.e., in total 9 parameters for PSE). Further, we fitted one slope parameter each for (a) unisensory auditory, (b) low-reliable visual, and (c) high-reliable visual conditions and (d) each audiovisual condition of the 2 visual reliability × 2 modality-specific report design (i.e., 7 slope parameters). Finally, as the conditions were presented in a randomized order, we fitted one single guess = lapse rate parameter across all conditions (i.e., one single parameter).

The Gaussians’ means and variances (σ^2^) of the unisensory conditions were used to compute the maximum likelihood predictions for the visual weights [*w_V_* in Eq. (1)] and the variance of the perceived signal location [σ*_AV_*^2^ in Eq. (3)]. The empirical visual weights [*w_V_*_,_*_emp_* in Eq. (2)] were computed from the audiovisual conditions with a small spatial cue conflict (i.e., ΔAV = 6° and = –6°). In the main analysis, the empirical audiovisual variances were computed jointly from the small cue conflict and congruent audiovisual conditions (see modeling constraints above).

In a follow-up analysis, we also obtained audiovisual variances selectively for the audiovisual congruent conditions by adding 4 independent slope parameters for the audiovisual congruent conditions (i.e., 21 parameters in total). As the small disparity trials were not included in the estimation of variance, this follow-up analysis allowed us to investigate whether modality-specific report can influence the integration process even for audiovisual congruent trials. In particular, we asked whether the audiovisual variance for the congruent conditions was immune to modality-specific report as predicted by the classic MLE model or depended on modality-specific report. We evaluated the MLE predictions using classic statistics and a Bayesian model comparison.

#### Classic statistics

In a model-based analysis, we compared the empirical visual weights and audiovisual variances with the MLE predictions and unisensory auditory and unisensory visual variances at the second (i.e., between-subject) random-effects level (Table 1). We used nonparametric Wilcoxon signed rank test to account for the small sample size (*n* = 5) and potential violations of normality assumptions.

**Table 1.** Statistical comparison of empirical weights (w__V,emp__) and standard deviations (σ_AV,emp_) obtained from the psychometric (behavior) and neurometric (fMRI) functions pertaining to the audiovisual conditions of high and low visual reliability with the MLE predictions (σ_AV,pred_, w_V,pred_) and unisensory standard deviations (σ__uniV__, σ__uniA__)

Comparison	VR+	VR–
w_V,emp_ – w_V,pred_		
Psychophysics	0.063	0.129
V1–V3	0.230	1
IPS0–4	0.631	1
Low-level auditory	0.064	1
σ_AV,emp_ – σ_AV,pred_		
Psychophysics	0.188	0.063
V1–V3	0.241	**0.001**
IPS0–4	**0.020**	0.275
Low-level auditory	1	1
σ_AV,emp_ – σ_uniV_		
Psychophysics	0.188	0.438
V1–V3	0.241	**0.001**
IPS0–4	**0.022**	1
Low-level auditory	0.883	0.963
σ_AV,emp_ – σ_uniA_		
Psychophysics	0.063	0.313
V1–V3	0.104	0.169
IPS0–4	**0.002**	0.086
Low-level auditory	1	1

Numbers denote *p* values (*p* < 0.05 printed in bold). Psychophysics parameters were compared using two-tailed Wilcoxon signed rank tests on individual parameters (random-effects analysis, df = 4). Neurometric parameters from V1–V3, IPS0–4, and low-level auditory regions were compared using a two-tailed bootstrap test (5000 bootstraps) on parameters computed across the sample (fixed-effects analysis). All comparisons of neurometric parameters were Bonferroni corrected across the three regions of interest. A, auditory; V, visual; VR±, high/low visual reliability.

In a model-free analysis, participant-specific visual weights and audiovisual variances were entered into second (i.e., between-subject) level analyses. At the random-effects level, we tested for the effects of visual reliability (high vs. low) and modality-specific report (visual vs. auditory) on the empirical visual weights and audiovisual variances using a 2 × 2 repeated measures ANOVA (Table 2). To account for the small sample size, we used a nonparametric procedure by computing the ANOVAs on rank-transformed empirical weights and variances (Conover and Iman, 1981). Further, we analyzed whether auditory signals biased visual reports and whether visual signals biased auditory reports by testing whether the visual weight was <1 or >0, respectively, while pooling over visual reliability. For these comparisons, we used one-sided Wilcoxon signed rank tests.

**Table 2.** Effects of visual reliability (VR), modality-specific report (MR), and their interaction (VR × MR) on empirical weights (w__V,emp__) and standard deviations (σ_AV,emp_) obtained from the psychometric (behavior) and neurometric (fMRI) functions

	w_V,emp_			σ_AV,emp_		
	VR	MR	VR × MA	VR	MR	VR × MR
Psychophysics (*F*, *p*)	5.149, 0.086	16.308, **0.016**	8.605, **0.043**	19.129, **0.012**	2.172, 0.215	18.892, **0.012**
V1-V3 (*p*)	0.346	0.131	0.957	**<0.001**	0.142	1
IPS0–4 (*p*)	**0.022**	**0.001**	1	**<0.001**	0.051	1
Low-level auditory (*p*)	0.693	0.217	1	1	0.419	1

Numbers denote *F* and *p* values for psychophysics parameters and *p* values for neurometric parameters (*p* < 0.05 printed in bold). Effects on psychophysics parameters were computed using a repeated measures ANOVA on rank-transformed weights and standard deviations (random-effects analysis, *n* = 5, df1 = 1, df2 = 4). Effects on neurometric parameters were computed using two-tailed bootstrap test (5000 bootstraps) on parameters computed across the sample (fixed-effects analysis). The analyses for neurometric weights and standard deviations were Bonferroni corrected across the three regions of interest.

As we employed a fixed-effects approach for the fMRI data to increase signal-to-noise ratio, in a follow-up analysis we applied the same fixed-effects approach to the behavioral data to ensure that differences between behavioral and fMRI results did not result from methodological differences. Unless otherwise stated, significance is reported at *p* < 0.05.

#### Bayesian model comparison

Using Bayesian model comparison analysis, we compared four models that manipulated whether visual reliability and modality-specific report could affect the PSEs and slopes of the audiovisual psychometric functions and whether their influence was predicted by the MLE model (Table 3). Model 1—null model: visual reliability and modality-specific report were not able to alter PSEs or slopes (i.e., integration of audiovisual signals with constant sensory weights irrespective of modality-specific report or reliability). Model 2—MLE model: visual reliability affected PSEs and slopes as predicted by MLE. Modality-specific report did not influence PSEs or slopes (again as predicted by MLE). Hence, we set the audiovisual PSEs and slopes to the MLE predictions based on the unisensory conditions as described in Eqs. (1) and (3). Model 3—reliability-weighted integration model: visual reliability influenced PSEs and slopes of the audiovisual conditions, yet not according to the MLE predictions. Hence, we allowed the PSEs and the slopes of the audiovisual conditions to differ across different reliability levels unconstrained by the MLE predictions. Yet, we did not allow top-down influences of modality-specific report to influence audiovisual PSEs or slopes. Model 4—full model: visual reliability and modality-specific report influenced both PSEs and slopes (i.e., the full model comparable to the analyses using classic statistics above).

**Table 3.** Results of the Bayesian model comparison between five competing models of the psychometric data

Model	I: Null model	II: MLE model	III: Reliability-weighting	IV: Full model
Parameters (*n*)	8	5	11	17
*R*^2^ (mean)	0.656	0.658	0.687	0.722
Relative BIC (sum)	0	59.662	1501.077	3202.034
Expected posterior *p*	0.111	0.111	0.111	0.667
Exceedance *p*	0.014	0.014	0.014	0.957
Protected exceedance *p*	0.026	0.026	0.026	0.921

I: In the null model, neither PSEs nor slopes depended on visual reliability or modality-specific report. II: In the MLE model, audiovisual PSEs and slopes were predicted based on unisensory variances as described in Eqs. (1) and (3). III: In the reliability-weighted integration model, PSEs and slopes depended on visual reliability unconstrained by MLE predictions. IV: In the full model, PSEs and slopes depended on visual reliability unconstrained by MLE predictions and modality-specific report (MR). *R*^2^, coefficient of determination, corrected for the binary response option (Nagelkerke, 1991). Relative BIC, Bayesian information criterion (i.e., an approximation to the model evidence) at the group level, i.e., subject-specific BICs summed over all subjects (BIC = *LL* − 0.5*M* × ln(*N*), where *LL* = log likelihood, *M* = number of parameters, *N* = number of data points) of a model relative to the null model (note that a greater relative BIC indicates that a model provides a better explanation of our data). Expected posterior *p*, probability that a given model generated the data for a randomly selected subject; exceedance *p*, probability that a given model is more likely than any other model; protected exceedance *p*, probability that one model is more likely than any other model beyond chance.

For all four models, psychometric functions were individually fitted to participants’ behavioral responses as described above. From the models’ log likelihood, we computed the Bayesian information criterion (BIC) as an approximation to the model evidence (Raftery, 1995). Bayesian model comparison (Stephan et al., 2009; Rigoux et al., 2014) was performed at the group level as implemented in SPM12 (Friston et al., 1994) based on the expected posterior probability (i.e., the probability that a given model generated the data for a randomly selected subject), the exceedance probability (i.e., the probability that a given model is more likely than any other model; Stephan et al., 2009), and the protected exceedance probability (additionally accounting for differences in model frequencies due to chance; Rigoux et al., 2014).

### MRI data acquisition

A 3T Siemens Magnetom Trio MR scanner was used to acquire both T1-weighted anatomic images and T2*-weighted axial echoplanar images (EPI) with BOLD contrast (gradient echo, parallel imaging using GRAPPA with an acceleration factor of 2, TR = 2480 ms, TE = 40 ms, flip angle = 90°, FOV = 192 × 192 mm, image matrix 78 × 78, 42 transversal slices acquired interleaved in ascending direction, voxel size = 2.5 × 2.5 × 2.5 mm + 0.25 mm interslice gap). In total, we acquired 353 volumes × 20 sessions for the audiovisual conditions, 353 volumes × 6–8 sessions for the unisensory conditions, 161 volumes × 2–4 sessions for the auditory localizer, and 159 volumes × 10–16 sessions for the visual retinotopic localizer (see below). This resulted in ∼18 h of scanning per participant assigned over 7–11 d. The first three volumes of each session were discarded to allow for T1 equilibration effects.

### fMRI data analysis: spatial ventriloquist paradigm

The fMRI data were analyzed with SPM8 (www.fil.ion.ucl.ac.uk/spm; Friston et al., 1994). Scans from each participant were corrected for slice timing, realigned, unwarped to correct for head motion, and spatially smoothed with a Gaussian kernel of 3-mm full width at half-maximum (de Beeck, 2010). The time series in each voxel was high-pass filtered to 1/128 Hz. All data were analyzed in native subject space. The fMRI experiment was modeled in an event-related fashion, with regressors entered into the design matrix after convolving each event-related unit impulse with a canonical hemodynamic response function and its first temporal derivative. In addition to modeling the 4 unisensory auditory, 8 unisensory visual, or 32 audiovisual conditions in a session, the general linear models (GLMs) included the realignment parameters as nuisance covariates to account for residual motion artifacts. The factor modality-specific report (visual vs. auditory) was modeled across sessions. The session-specific parameter estimates pertaining to the canonical hemodynamic response function (HRF) defined the magnitude of the BOLD response to the unisensory or audiovisual stimuli in each voxel.

To apply the MLE analysis approach to spatial representations at the neural level, we first extracted the parameter estimates pertaining to the HRF magnitude for each condition and session from voxels of regions defined in separate auditory and retinotopic localizer experiments (see below). This yielded activation patterns from the unisensory auditory and visual conditions and the audiovisual congruent (ΔAV = 0°) and small spatial cue conflict (ΔAV ± 6°) conditions. All activation patterns (i.e., from each condition in each session) were *z*-normalized across all voxels of a region of interest to avoid the effects of regionwide activation differences between conditions. We then trained a linear support vector classification model (as implemented in LIBSVM 3.14; Chang and Lin, 2011) to learn the mapping from activation patterns from the audiovisual congruent conditions to the categorical left vs. right location of the audiovisual signal in a subject-specific fashion. Importantly, we selectively used activation patterns from audiovisual congruent conditions from all but one audiovisual session for support vector classification training (i.e., training was done across sessions of auditory and visual report). The trained support vector classification model was then used to decode the signal location (left vs. right) from the activation patterns of the spatially congruent and conflicting audiovisual conditions of the remaining audiovisual session. Hence, given the learned mapping from audiovisual activation patterns of the congruent conditions to the true left-versus-right stimulus location class, the support vector classifier decoded the stimulus location for activation patterns elicited by the audiovisual spatially small conflict trials. In a leave-one-out cross-validation scheme, the training-test procedure was repeated for all audiovisual sessions. Finally, the support vector classification model was trained on audiovisual congruent conditions from all audiovisual sessions and then decoded the categorical signal location (left vs. right) from activation patterns of the separate unisensory auditory and visual sessions.

In line with our behavioral analysis, we plotted the fraction of decoded “right” as a function of the unisensory signal location for the unisensory auditory and visual conditions (Fig. 3*E*). For the audiovisual spatially congruent and small cue conflict conditions, we plotted the fraction of decoded “right” as a function of the mean signal location of the true auditory and visual signal locations (separately for auditory/visual report × visual reliability levels; Fig. 3*A–D*). Because of the lower signal-to-noise ratio of fMRI data, we fitted cumulative Gaussians as neurometric functions to the fraction decoded “right” pooled (i.e., averaged) across all participants (i.e., fixed-effects analysis). To obtain empirical and MLE predicted weights and variances, we employed the same procedure and equations as explained in the section Behavioral data. Confidence intervals for empirical and predicted weights and variances were computed using Palamedes’ parametric bootstrap procedure (1000 bootstraps).

In the model-based analysis, we used two-tailed bootstrap tests (5000 bootstrap samples; Efron and Tibshirani, 1994) to investigate whether empirical sensory weights and variances for audiovisual conditions were significantly different from the MLE predictions. Further, we assessed whether variances for audiovisual conditions were significantly different from variances for unisensory conditions (Table 1). For these model-based analyses, we parametrically bootstrapped the fraction of decoded “right” and in turn fitted neurometric functions to the bootstrapped data. From the bootstrapped auditory, visual, and audiovisual psychometric functions, we generated bootstrap distributions of MLE predictions for the sensory weights and variances and their empirical counterparts. Bootstrapped null-distributions for a specific parameter comparison (e.g., predicted weight vs. empirical weight) were generated by computing the difference between predicted and empirical parameters (e.g., predicted weight vs. empirical weight) for each bootstrap and subtracting the observed original difference (Efron and Tibshirani, 1994). From this bootstrapped null-distribution, the two-tailed significance of a parameter comparison was computed as the fraction of bootstrapped absolute values that were greater or equal to the observed original absolute difference [e.g., violation of MLE prediction: abs(w_V,predicted,original_ – w_V,empirical,original_)]. Absolute values were used to implement a two-tailed test (Efron and Tibshirani, 1994). Violations of MLE predictions were tested across modality-specific report because the MLE model does not predict a modulation by report (i.e., mean and variance parameters of the psychometric functions were held constant across levels of modality-specific report).

Similarly, in the model-free analysis, we used two-tailed bootstrap tests (5000 bootstrap samples) to analyze the effects of visual reliability (high vs. low), modality-specific report (visual vs. auditory), and their interaction on the empirical visual weights and audiovisual variances (Table 2). Bootstrapped null-distributions of weights and audiovisual variances for each of the four conditions in our modality-specific report (visual vs. auditory) × visual reliability (high vs. low) design were generated by computing the contrast value of interest (e.g., high minus low visual reliability) for the sensory weights or variances for each bootstrap and subtracting the corresponding contrast value obtained from the original data (Efron and Tibshirani, 1994). From this bootstrapped null-distribution, the two-tailed significance (against zero) of the effects of interest (e.g., high vs. low reliability) was computed as the fraction of bootstrapped absolute contrast values that were greater or equal to the observed original absolute contrast value. Mean and variance parameters of the psychometric functions were set to be equal across the levels of modality-specific report to test selectively for the main effect of visual reliability. Conversely, mean and variance parameters of the neurometric functions were set to be equal across levels of visual reliability to test selectively for the main effect of modality-specific report. By contrast, mean and variance parameters of the neurometric functions varied across levels of visual reliability and modality-specific report to test for the interaction effect of modality-specific report and visual reliability. For all analyses reported in Tables 1 and 2, we report *p* values corrected for multiple comparisons across the three regions of interest using a Bonferroni correction.

Finally, we investigated whether multisensory influences can be observed already at the primary cortical level during (1) audiovisual or (2) even unisensory (i.e., auditory or visual) stimulation. (1) To assess cross-modal influences during audiovisual stimulation, we computed a one-sided bootstrap test (5000 bootstrap samples) by fitting neurometric functions to bootstrapped data (see above) averaged across visual reliability and modality-specific report. Specifically, we tested whether the empirical weight pertaining to the visual signal was <1 (indicating auditory influence) in visual regions and whether it was >0 (indicating visual influence) in auditory regions. (2) To assess cross-modal influences during unisensory stimulation, we tested whether the slope (i.e., the perceptual threshold 1/σ) of the neurometric functions was significantly >0 in unisensory conditions. As we were interested only in whether the slope was significantly >0 (rather than the exact size), we used a constrained approach by fitting neurometric functions to auditory stimulation data in visual cortex and to visual stimulation data (pooled over visual reliability levels) in auditory cortex with lapse and guess rates set to 0. We determined whether a slope parameter was significantly larger than zero using a one-tailed bootstrap test (5000 bootstrap samples). Across all analyses, we confirmed the validity of the bootstrap tests in simulations showing that simulated *p* values converged to a nominal α level of 0.05 under the null hypothesis.

### Control analyses to account for motor preparation and global activation differences between hemispheres

To account for activations related to motor planning (Andersen and Buneo, 2002), a first control analysis included the trial-wise button responses as a nuisance covariate into the first-level GLM (i.e., one regressor for each of the four response buttons). We then repeated the multivariate decoding analysis using activation patterns from intraparietal sulcus (IPS0–4, see below) where motor responses were explicitly controlled (Fig. 3-1).

10.1523/ENEURO.0315-17.2018.f3-1 Figure 3-1 fMRI results in the intraparietal sulcus when controlling for motor responses: neurometric functions, visual weights and audiovisual variances. In intraparietal sulcus (IPS0–4), neurometric functions were fitted to the fraction of decoded “right” location responses plotted as a function of the mean audiovisual (AV) location (see figure 2 legend for additional information). To control for motor planning in IPS0–4, activation patterns were obtained from a general linear model that modeled participants’ trial-wise button responses as a nuisance variable. ***A–D***, Neurometric functions are plotted separately for the four conditions in our 2 (visual reliability: high, VR+ vs. low, VR-) x 2 (modality-specific report: auditory vs. visual) factorial design. ***E***, In unisensory conditions, psychometric functions were fitted to the fraction of right location responses plotted as a function of the signal location from unisensory auditory (A) and visual conditions of high (V, VR+) and low (V, VR-) visual reliability. ***F***, Visual weights (mean and 68% bootstrapped confidence interval): MLE predicted and empirical visual weights for 2 (visual reliability: high, VR+ vs. low, VR-) x 2 (modality-specific report: auditory vs. visual) AV conditions. To facilitate the comparison with the MLE predictions that do not depend on modality-specific report, the visual weights are also plotted after pooling the data across both report conditions and re-fitting the neurometric functions. ***G***, Standard deviations (σ, mean and 68% bootstrapped confidence interval): Unisensory and audiovisual MLE predicted and empirical standard deviations for the same combination of conditions as in ***F***. For illustrational purposes standard deviations were normalized by the auditory standard deviation. Download Figure 3-1, TIF file.

Given the contralateral encoding of space in visual (Wandell et al., 2007) and auditory (Ortiz-Rios et al., 2017) regions, a second control analysis evaluated the impact of global activation differences between hemispheres on the classifier’s performance. In this control analysis, we *z*-normalized the activation patterns separately for voxels of the left and right hemisphere in each condition before multivariate decoding (Figs. 3-2 and 4–1). In other words, multivariate decoding was applied to activation patterns where global activation differences between hemispheres were removed.

10.1523/ENEURO.0315-17.2018.f3-2 Figure 3-2 fMRI results in the intraparietal sulcus when controlling for global interhemispheric activation differences: neurometric functions, visual weights and audiovisual variances. In intraparietal sulcus (IPS0–4), neurometric functions were fitted to the fraction of decoded “right” location responses plotted as a function of the mean audiovisual (AV) location (see figure 2 legend for additional information). To control for global interhemispheric activation differences, activation patterns were z normalized separately for the left and right hemisphere within each condition prior to multivariate decoding. ***A–D***, Neurometric functions are plotted separately for the four conditions in our 2 (visual reliability: high, VR+ vs. low, VR-) x 2 (modality-specific report: auditory vs. visual) factorial design. ***E***, In unisensory conditions, psychometric functions were fitted to the fraction of right location responses plotted as a function of the signal location from unisensory auditory (A) and visual conditions of high (V, VR+) and low (V, VR-) visual reliability. ***F***, Visual weights (mean and 68% bootstrapped confidence interval): MLE predicted and empirical visual weights for 2 (visual reliability: high, VR+ vs. low, VR-) x 2 (modality-specific report: auditory vs. visual) AV conditions. To facilitate the comparison with the MLE predictions that do not depend on modality-specific report, the visual weights are also plotted after pooling the data across both report conditions and re-fitting the neurometric functions. ***G***, Standard deviations (σ, mean and 68% bootstrapped confidence interval): Unisensory and audiovisual MLE predicted and empirical standard deviations for the same combination of conditions as in ***F***. For illustrational purposes standard deviations were normalized by the auditory standard deviation. Download Figure 3-2, TIF file.

10.1523/ENEURO.0315-17.2018.f4-1 Figure 4-1 fMRI results in low-level visual and auditory regions when controlling for interhemispheric activation differences: Visual weights and audiovisual variances. To control for interhemispheric activation differences, activation patterns were z normalized separately in the left and right hemisphere within each condition prior to multivariate pattern decoding. ***A***, Visual weights (mean and 68% bootstrapped confidence interval): MLE predicted and empirical visual weights for 2 (visual reliability: high, VR+ vs. low, VR-) x 2 (modality-specific report: auditory vs. visual) audiovisual conditions in low-level visual regions (V1-3). To facilitate the comparison with the MLE predictions that do not depend on modality-specific report, the visual weights are also plotted after pooling the data across both report conditions and re-fitting the neurometric functions. ***B***, Standard deviations (σ, mean and 68% bootstrapped confidence interval): Unisensory and audiovisual MLE predicted and empirical standard deviations for the same combination of conditions as in ***A***. For illustrational purposes standard, deviations were normalized by the auditory standard deviation. ***C***, Visual weights (mean and 68% bootstrapped confidence interval): MLE predicted and empirical visual weights in low-level auditory regions (hA) as shown in ***A***. ***D***, Standard deviations (σ, mean and 68% bootstrapped confidence interval): Unisensory and audiovisual MLE predicted and empirical standard deviations of spatial representations in low-level auditory regions (hA) as shown in ***B***; note that the upper confidence interval for the visual variance is truncated for illustrational purposes. For illustrational purposes, standard deviations were normalized by a combined visual standard deviation for low and high visual reliability. Download Figure 4-1, TIF file.

### Effective connectivity analyses

Using dynamic causal modeling (DCM), we investigated the modulatory effects of visual reliability on the effective connectivity from early visual regions to IPS and modality-specific report on the connectivity from prefrontal cortex (PFC) to IPS. For each subject, we constructed four bilinear DCMs (Friston et al., 2003). Each DCM included four regions: low-level visual regions (V1–3), low-level auditory regions, IPS0–4, and PFC. Low-level visual and auditory regions and IPS0–4 were defined functionally as described in Auditory and visual retinotopic localizer. PFC was defined anatomically for each individual as the middle frontal gyrus based on the anatomic cortical parcellation of the Desikan–Killiany atlas (Desikan et al., 2006) implemented in Freesurfer 5.1.0 (Dale et al., 1999). Region-specific time series comprised the first eigenvariate of activations across all voxels within each region that were significant at *p* < 0.001 in the effects-of-interest contrast across all conditions in the first-level within-subject GLMs (*F* test, uncorrected).

**Figure 5.**
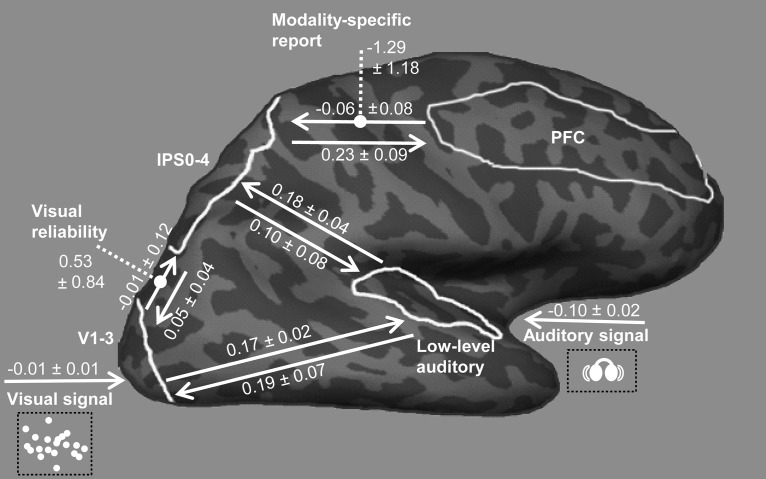
Dynamic causal modeling. In the optimal model (i.e., the model with the highest exceedance probability), visual reliability modulated the connection from V1-3 to IPS0–4 and modality-specific report modulated the connection from PFC to IPS0–4. Values are across-subjects means (± SEM) indicating the strength of extrinsic, intrinsic and modulatory connections. The modulatory effects quantify how visual reliability and modality-specific report change the values of intrinsic connections.

In all DCM models, V1-3, IPS0–4, and low-level auditory regions were bidirectionally connected, and PFC was bidirectionally connected to IPS0–4 (i.e., intrinsic connectivity structure; Fig. 5). Synchronous audiovisual signals entered as extrinsic input into V1–3 and low-level auditory regions. Holding intrinsic and extrinsic connectivity structure constant, the 2 × 2 candidate DCMs factorially manipulated the presence/absence of the following modulatory effects: (a) visual reliability on V1–3 → IPS0–4 (on vs. off) and (b) modality-specific report on PFC → IPS0–4 (on vs. off). After fitting the full model, which included both modulatory effects, to the fMRI data of each subject, we used Bayesian model reduction to estimate the model evidences and parameters of the reduced models (Friston et al., 2016). To determine the most likely of the four DCMs given the observed data from all subjects, we implemented a fixed-effects (Penny et al., 2004) and a random-effects (Stephan et al., 2009) group analysis. The fixed-effects group analysis was implemented by taking the product of the subject-specific Bayes factors over subjects (this is equivalent to the exponentiated sum of the log model evidences of each subject-specific DCM; Penny et al., 2004). Because the fixed-effects group analysis can be distorted by outlier subjects, Bayesian model comparison was also implemented in a random-effects group analysis. At the random-effects level, we report the expected posterior probability, the exceedance probability, and the protected exceedance probability (Stephan et al., 2009; Rigoux et al., 2014; Table 4).

**Table 4.** Results of the model comparison of the 2 × 2 dynamic causal models in which visual reliability (VR) modulated the connection from V1-3 to IPS0–4 and modality-specific report (MR) modulated the connection from PFC to IPS0–4

Factor	Modulation VR and MR	Modulation VR	Modulation MR	No modulation
Model evidence (FFX)	0	–52.947	–54.033	–90.45
Posterior *p* (FFX)	1	0	0	0
Expected posterior *p* (RFX)	0.587	0.136	0.139	0.139
Exceedance *p* (RFX)	0.902	0.032	0.033	0.033
Protected exceedance *p* (RFX)	0.699	0.1	0.101	0.101

FFX, fixed-effects analysis; RFX, random-effects analysis; model evidence corresponds to free energy (relative to full model) summed over participants (i.e., larger is better); expected posterior *p*, probability that a given model generated the data for a randomly selected subject; exceedance *p*, probability that a given model is more likely than any other model; protected exceedance *p*, probability that one model is more likely than any other model beyond chance.

### Auditory and visual retinotopic localizer

Regions of interest along the auditory and visual processing hierarchies were defined in a subject-specific fashion based on auditory and visual retinotopic localizers. In the auditory localizer, participants were presented with brief bursts of white noise at –10° or 10° angle (duration 500 ms, stimulus onset asynchrony 1 s). In a one-back task, participants indicated via a key press when the spatial location of the current trial was different from the previous trial; 20 s blocks of auditory stimulation (i.e., 20 trials) alternated with 13 s of fixation periods. The auditory locations were presented in a pseudorandomized fashion to optimize design efficiency. Similar to the main experiment, the auditory localizer sessions were modeled in an event-related fashion. Auditory-responsive regions were defined as voxels in superior temporal and Heschl’s gyrus showing significant activations for auditory stimulation relative to fixation (*t* test, *p* < 0.05, family-wise error corrected). Within these regions, we defined primary auditory cortex (A1) based on cytoarchitectonic probability maps (Eickhoff et al., 2005) and referred to the remainder (i.e., planum temporale and posterior superior temporal gyrus) as higher-order auditory cortex (hA).

Visual regions of interest were defined using standard phase-encoded retinotopic mapping (Sereno et al., 1995). Participants viewed a checkerboard background flickering at 7.5 Hz through a rotating wedge aperture of 70° width (polar angle mapping) or an expanding/contracting ring (eccentricity mapping). The periodicity of the apertures was 42 s. Visual responses were modeled by entering a sine and cosine convolved with the hemodynamic response function as regressors into the design matrix of the general linear model. The preferred polar angle (or eccentricity, respectively) was determined as the phase lag for each voxel by computing the angle between the parameter estimates for the sine and the cosine. The phase lags for each voxel were projected on the reconstructed, inflated cortical surface using Freesurfer 5.1.0 (Dale et al., 1999). Visual regions V1–V3 and IPS0–4 were defined as phase reversal in angular retinotopic maps. IPS0–4 were defined as phase reversal along the anatomic IPS resulting in contiguous, approximately rectangular regions (Swisher et al., 2007).

For the decoding analyses, the auditory and visual regions were combined from the left and right hemisphere. Support vector classification training was then applied separately to activation patterns from each region. To improve the signal-to-noise ratio when fitting neurometric functions (see Figs. 3 and 4), the decoded signal sides (right vs. left) from low-level visual regions (V1–3), intraparietal sulcus (IPS0–4), and low-level auditory regions (A1, hA) were pooled. Additional analyses showed similar audiovisual spatial integration within these three regions (Rohe and Noppeney, 2015a, 2016).

## Results

### Spatial ventriloquist paradigm

In the fMRI study, participants were presented with auditory, visual, and audiovisual signals sampled randomly from four possible spatial locations along the azimuth (–10°, –3.3°, 3.3°, or 10°; Fig. 1). Audiovisual signals included in this study were either spatially congruent (ΔAV = 0°) or incongruent with a small spatial conflict (ΔAV = ±6°). The reliability of the visual signal was either high or low. Modality-specific report was manipulated by instructing participants to report the location of either the visual or the auditory signal component during the audiovisual conditions.

Figs. 2 and 3 present the psychometric functions estimated from the behavioral button responses after categorization into “left” or “right” responses and the neurometric functions estimated from spatial locations (left vs. right) decoded from fMRI responses. The psychometric (respective to neurometric) functions show the fraction of “right” responses as a function of the mean signal location for each condition. If the visual reliability is greater than the auditory reliability (i.e., visual weight > 0.5), we would expect the function to be shifted toward the right for a positive spatial conflict (A-V = ΔAV = +6°, i.e., the visual signal is presented 6° to the left of the auditory signal) and to the left for a negative spatial conflict (ΔAV = –6°, i.e., the visual signal is presented 6° to the right of the auditory signal). As a consequence, the point of subjective equality (PSE, defined by the abscissa’s value for 50% proportion “right” responses) of the psychometric functions for the spatial conflict conditions can be employed to compute the empirical sensory weights for the different conditions (Ernst and Banks, 2002; Fetsch et al., 2012).

In short, we (1) fitted psychometric or neurometric functions to unisensory, audiovisual congruent, and small spatial conflict conditions and (2) derived the sensory weights from the psychometric/neurometric functions (i.e., shift in PSE) of the conflict conditions and derived the variances from the psychometric/neurometric functions of the spatial conflict and congruent conditions (Figs. 2*A–D* and 3*A–D* ). In a model-based analysis, we compared the sensory weights (Figs. 2*F*, 3*F*, and 4*A*,*C* ) and variances (Figs. 2*G*, 3*G*, and 4*B*,*D* ) with MLE predictions that were derived from the unisensory conditions (Figs. 2*E* and 3*E* ). Because MLE predictions do not depend on modality-specific report, we compared the MLE predictions with the empirical sensory weights and variances while pooling over visual and auditory report.

For both behavioral and neural data, we addressed two questions. First, in a model-based analysis using classic statistics, we investigated whether the MLE predictions that were derived from the unisensory conditions were in line with the empirical sensory weights computed from the audiovisual spatial conflict conditions and the variances computed either from the audiovisual conflict and congruent conditions or from the congruent conditions alone. Second, in a model-free analysis using classic statistics, we investigated whether the empirical sensory weights and variances were influenced by visual reliability or modality-specific report. For the psychophysics data, we also addressed these two questions using Bayesian model comparison to formally compare the MLE model to alternative models that do or do not allow visual reliability and modality-specific report to influence the PSEs (i.e., Gaussian means) and/or slopes (i.e., Gaussian variances) of the audiovisual conditions.

### Psychophysics results

#### Classic statistics

In the model-based MLE analysis, the slopes of the psychometric functions for the unisensory conditions indicated that for high visual reliability, the visual representations were more reliable than the auditory representations (Fig. 2*E*). By contrast, for low visual reliability, the variances obtained from the auditory and visual psychometric functions were comparable.

The visual weights obtained from the audiovisual conflict conditions were approximately in line with the MLE predictions derived from those unisensory psychometric functions, although there was a nonsignificant difference between predicted and empirical visual weights for high visual reliability (Fig. 2*F*; Table 1). Moreover, although the variance of the perceived signal location was significantly reduced relative to the unisensory auditory condition in case of high visual reliability (Fig. 2*G*; Table 1), it was not reduced relative to the variances obtained for the most reliable unisensory condition. In particular for the low visual reliability conditions where auditory and visual reliabilities were approximately matched, we did not observe a substantial variance reduction as predicted by MLE (Fig. 2*G*, i.e., a marginally significant difference between MLE predictions and empirical audiovisual variances for low visual reliability; Table 1).

For the model-free analysis, the visual weights were marginally greater for high relative to low visual reliability (Table 2). Yet, contrary to the MLE predictions, we also observed a significant effect of modality-specific report on the visual weights. Visual weights were greater for visual relative to auditory report. For visual report, the visual weight was not significantly <1 (*p* = 0.219, one-sided Wilcoxon signed rank test pooling across visual reliability), indicating that the auditory signal did not significantly influence visual location reports. For auditory report, the visual weight was significantly >0 (*p* = 0.032), indicating that the visual signal “attracted” auditory location reports, known as the ventriloquist effect (Radeau and Bertelson, 1977). Most importantly, we observed a significant interaction between reliability and modality-specific report (Table 2). The interaction arose from the top-down influences of modality-specific report being more pronounced for the low visual reliability conditions when the auditory and visual reliabilities were approximately matched. Indeed, for low visual reliability conditions, the psychometric functions of the cue conflict conditions were shifted toward the true visual location for visual report (Fig. 2*D*) but toward the true auditory location during auditory report (Fig. 2*B*). By contrast, for high visual reliability conditions, the psychometric functions of the cue conflict conditions were shifted toward the true visual location for both auditory and visual report (Fig. 2*A*,*C*).

The variance of the perceived signal location was significantly influenced by visual reliability (Table 2), but not by modality-specific report. Critically, we observed a significant interaction between the two factors. The significant interaction resulted from the effect of modality-specific report being revealed predominantly for high visual reliability, but not for low visual reliability, when the auditory and visual reliabilities were approximately matched. The results suggest that the variance of the perceived signal location was influenced predominantly by the sensory modality that needed to be attended and reported. In other words, participants did not fuse sensory signals into one unified percept. Instead, modality-specific report increased the influence of the reported signal in the final percept. Importantly, the interaction effect was also observed when we estimated the audiovisual variance selectively from the audiovisual congruent conditions (interaction of visual reliability and modality-specific report: *F*_1,4_ = 34.507, *p* = 0.004; effect of visual reliability: *F*_1,4_ = 23.721, *p* = 0.008). The results confirm that modality-specific report can selectively increase the influence of the reported sensory signal on the perceived signal location under classic forced-fusion conditions where sensory signals co-occur in space and time. If observers report the auditory location, the variance is determined predominantly by the variance of the auditory signals (and vice versa for visual report).

#### Bayesian model comparison

In line with the results from classic statistics, the formal Bayesian model comparison demonstrated that the MLE model was not the best model of our data. Instead, the strongest model evidence was observed for a model in which visual reliability and modality-specific report influenced the PSE and slope parameters unconstrained by MLE predictions (i.e., protected exceedance probability = 0.916; Table 3). Critically, the model evidence combines an accuracy (i.e., model fit) and a complexity term that penalizes complex models with more free parameters. For instance, the MLE model is very parsimonious, with only 5 parameters, whereas the winning model includes 17 free parameters. Our results thus suggest that modeling effects of reliability and modality-specific report are critical to account for an observer’s localization responses.

#### Summary

Collectively, our psychophysics results suggest that auditory and visual signals were integrated approximately weighted by their relative reliabilities. However, the weights were not assigned solely in proportion to the relative bottom-up sensory reliabilities, as predicted by the MLE model, but were also modulated by modality-specific report and potentially associated attentional processes. The visual weight was greater when the location of the visual signal was attended and reported. Likewise, the variance of the perceived signal location depended on modality-specific report. Hence, irrespective of whether the audiovisual signals were congruent or in small spatial conflict, participants did not integrate them into one unified percept as predicted by MLE. Instead, they were able to selectively control the influence of auditory or visual signal components depending on task instructions. As a result, observers did not significantly benefit from audiovisual stimulation: there was no reduction in variance of the perceived signal location relative to the most reliable unisensory percept as predicted by MLE optimal integration.

### fMRI results

To investigate the neural processes by which human observers integrate sensory signals into spatial representations, we decoded spatial information from fMRI activation patterns. The patterns were extracted from low-level visual regions (V1–3), low-level auditory regions (primary auditory cortex and planum temporale), and intraparietal sulcus (IPS0–4). We trained a support-vector classification model on fMRI activation patterns selectively from audiovisual congruent conditions (ΔAV = 0°) to learn the mapping from activation patterns to the signal location label (left vs. right). The trained model then decoded the signal location class (left vs. right) from activation patterns in audiovisual spatial conflict conditions (ΔAV = ±6°) as well as unisensory auditory and visual conditions. The decoded signal location class, i.e., the “left/right location response” given by a particular brain area, was then analyzed using the same procedures that were applied to the categorized (left vs. right) behavioral location responses (see above).

#### Visual regions

##### Auditory influences under unisensory auditory stimulation

In line with previous reports of multisensory influences at the primary cortical level (Meyer et al., 2010; Liang et al., 2013; Vetter et al., 2014), we observed a significant positive slope of the psychometric function estimated for the unisensory auditory conditions in low-level visual areas (*p* < 0.001, one-sided bootstrap test). These results indicate that auditory signals (when presented in isolation) elicit spatial representations in low-level visual regions (V1–3). Yet, going beyond previous studies (Meyer et al., 2010; Liang et al., 2013; Vetter et al., 2014), our results demonstrate that these auditory influences on visual cortex (in the absence of concurrent visual signals) are rather limited and induce only unreliable representations compared with the spatial representations decoded under unisensory visual stimulation (see visual and auditory variance obtained for the neurometric functions under unisensory stimulation in low-level visual areas; Fig. 4*B*).

##### Model-based MLE analysis

Based on those unisensory visual and auditory neurometric functions, MLE predicted negligible auditory influences on spatial representations during audiovisual stimulation (Fig. 4*A*). Indeed, in line with those MLE predictions, the representations formed from audiovisual signals relied predominantly on visual input, as indicated by a visual weight that did not significantly deviate from 1 (*p* = 0.818, one-sided bootstrap test pooling the visual weight across conditions). Moreover, in line with MLE predictions, the variance of the audiovisual representations was comparable to unisensory visual variances (Fig. 4*B* and Table 1).

##### Model-free analysis

The sensory weights were not significantly modulated by visual reliability or modality-specific report (Table 2). Yet the audiovisual variance was smaller for high versus low visual reliability, indicating that the representations under audiovisual stimulation are predominantly determined by the visual signals and hence depend solely on the reliability of the visual signal.

#### Auditory regions

##### Visual influences under unisensory visual stimulation

In parallel to our findings in visual regions, the slope of the neurometric functions estimated from the unisensory visual conditions was again significantly positive, indicating that visual signals alone elicit spatial representations in auditory areas (*p* = 0.004, one-sided bootstrap test pooling across visual reliability). Yet, when compared to the spatial representations decoded under unisensory auditory stimulation, these visual influences on auditory cortex (in the absence of concurrent auditory signals) were rather limited and induced only unreliable representations (see visual and auditory variance obtained from the neurometric functions under unisensory stimulation in low-level auditory areas; Fig. 4*D*).

##### Model-based MLE analysis

Based on those unisensory variances, the MLE model predicted a visual weight close to 0 (Fig. 4*C*) and an audiovisual variance approximately identical to the auditory variance for the audiovisual conditions irrespective of visual reliability or modality-specific report (Fig. 4*D*). Although we did not observe any significant deviations from the MLE predictions, the empirical visual weight was greater than predicted by MLE. This was particularly pronounced for high visual reliability conditions. Fig. 4*C* reveals that this deviation emerged predominantly for conditions when the visual signal needs to be attended and reported. These findings may be explained by cross-modal attentional top-down effects operating from vision to audition. Indeed, the visual weight was significantly >0 (*p* = 0.004; one-sided bootstrap test pooling the visual weight across conditions), indicating that visual signals exerted a stronger influence on auditory areas during audiovisual stimulation than vice versa (see above: the visual weight was not significantly lower than one in visual regions).

##### Model-free analysis

We did not observe an effect of visual signal reliability or modality-specific report or an interaction between the two factors on the visual weight or variance estimated from the audiovisual conditions in auditory regions (Table 2).

#### Parietal areas

##### Model-based MLE analysis

In IPS0–4, the neurometric functions for the unisensory conditions indicated that the neural representations for unisensory visual signals were more reliable than those for unisensory auditory signals at both levels of signal reliability (Fig. 3*E*). This greater reliability of visual IPS representations is consistent with the well-established visual dominance of IPS (Swisher et al., 2007; Wandell et al., 2007). Based on these unisensory variances, MLE predicted a visual weight that was close to 1 for high visual reliability and decreased for low visual reliability. Indeed, the visual weights estimated from the audiovisual conditions were approximately in accordance with these MLE predictions (Fig. 3*F* and Table 1). By contrast, the empirical audiovisual variance was in line with the MLE predictions only for low visual reliability conditions, but significantly smaller than MLE predictions for high visual reliability conditions (Fig. 3*G* and Table 1). This surprising result needs to be further investigated and replicated in future studies.

##### Model-free analysis

In IPS0–4, the visual weight and the audiovisual variance were modulated by visual reliability and modality-specific report (Table 2). IPS0–4 integrated audiovisual signals depending on bottom-up visual reliability and top-down effects of modality-specific report approximately in line with the profile of the behavioral weights (Fig. 3*F*). Likewise, the audiovisual variance was reduced for high relative to low visual reliability conditions. Moreover, modality-specific report also marginally influenced the variance of the spatial representation obtained from the audiovisual conditions. The variance for the audiovisual conditions was smaller for auditory than visual report (note that this marginally significant modulation of variance by modality-specific report was also observed when the analysis focused selectively on the audiovisual spatially congruent conditions, *p* = 0.096). The smaller variance for auditory relative to visual report in IPS contrasts with the variance reduction under visual report observed at the behavioral level (note that this difference cannot be explained by methodological differences, because we observed comparable results when applying a fixed-effects analysis at the behavioral level). Potentially this neurobehavioral dissociation can be explained by the fact that the auditory report conditions were more difficult and engaged more attentional resources, thereby leading to an increase in reliability of BOLD-activation patterns. Most importantly, however, both behavioral and neural data provide convergent evidence that the sensory weights and to some extent the variances—even for audiovisually congruent trials—depend on both bottom-up visual reliability and top-down effects of modality-specific report.

### Control analyses: eye movements, motor planning, and interhemispheric activation differences

No significant differences in eye movement indices (percentage saccades, percentage eye blinks, poststimulus mean horizontal eye position) were observed across any audiovisual conditions [see the supplemental results reported in Rohe and Noppeney (2016)]. For the unisensory visual conditions, we observed only a small significant effect of the visual signal location on the poststimulus mean horizontal eye position (*F*_3,9_ = 4.9, *p* = 0.028). However, this effect did not depend on the reliability of the visual signal.

Further, a control analysis that decoded IPS activation patterns from a GLM that accounted for participants’ trial-wise button responses revealed results for sensory weights and audiovisual variances (Fig. 3-1) highly similar to our initial analysis. These results suggest that IPS represents audiovisual spatial representations that cannot be completely attributed to motor planning and response selection.

Finally, given the predominantly contralateral representations of the peri-personal space in visual (Wandell et al., 2007) and auditory regions (Ortiz-Rios et al., 2017), we investigated the impact of global activation differences between the left and right hemispheres on classification performance. When we removed interhemispheric activation differences from activation patterns before decoding, we found comparable results for sensory weights and audiovisual variances (Figs. 3-2 and 4-1). Thus, audiovisual spatial representations are encoded in hemisphere-specific activation patterns that go beyond differences in global signal across hemispheres in visual and auditory regions.

### Dynamic causal modeling

Our multivariate pattern analysis showed that visual reliability and modality-specific report influenced visual weights and audiovisual variances in IPS0–4. Using DCM and Bayesian model comparison, we next investigated whether these influences were mediated by modulatory effects of reliability on effective connectivity from V1–3 to IPS0–4 and modality-specific report on connectivity from PFC to IPS0–4 (Fig. 5). PFC potentially mediates the effect of modality-specific report because PFC exerts top-down control on sensory processing (Noudoost et al., 2010; Zanto et al., 2011) by changing the connectivity to parietal regions (Buschman and Miller, 2007). Indeed, in the winning model, visual reliability modulated the connection from V1–3 to IPS0–4, and modality-specific report modulated the connection from PFC to IPS0–4 (i.e., protected exceedance probability = 0.699; Table 4).

## Discussion

The classic MLE model assumes that auditory and visual signals that arise from a common source are integrated weighted by their sensory reliabilities into one unified representation. Critically, the sensory weights are thought to be determined solely by the reliabilities of the sensory signals and immune to task-dependent top-down control. Indeed, abundant evidence suggests that human observers can combine signals within and across the senses near-optimally as predicted by the MLE model (Jacobs, 1999; Ernst and Banks, 2002; van Beers et al., 2002; Knill and Saunders, 2003; Alais and Burr, 2004; Hillis et al., 2004; Saunders and Knill, 2004; Rosas et al., 2005; but see Battaglia et al., 2003). Although the forced-fusion assumption of a common signal cause usually holds for integration within a sensory modality (Hillis et al., 2002), it is often violated when integrating signals across sensory modalities (Gepshtein et al., 2005; Parise et al., 2012). For example, it remains controversial whether multisensory integration and, more specifically, the sensory weights can be modulated by top-down control (Helbig and Ernst, 2008; Talsma et al., 2010; Vercillo and Gori, 2015).

The present study investigated the extent to which audiovisual spatial signals are integrated in line with the quantitative predictions of the MLE model at the behavioral and neural levels. Importantly, although many previous MLE studies (Battaglia et al., 2003; Alais and Burr, 2004) presented only signals with a small conflict and asked participants to report the location of the “audiovisual stimulus”, thereby encouraging integration of signals into one unified percept, we instructed participants to attend and report either the visual or the auditory signals (Stein et al., 1989; Wallace et al., 2004; Körding et al., 2007). Thus, our task instructions pushed observers to focus selectively on one signal component rather than treating the two signals as originating necessarily from one common object.

At the behavioral level, our results demonstrate that observers did not integrate signals into one unified percept as predicted by MLE. The visual weight increased not only when the visual signal was more reliable but also when it needed to be reported. Most importantly, even when auditory and visual signals were spatially congruent, i.e., likely to originate from one single object, observers were able to focus selectively on one sensory modality as indicated by differences in variance for the spatial representations obtained from auditory and visual report conditions. In other words, modality-specific attention and report modulated the sensory weights not only during the spatial conflict conditions but also during the congruent conditions. Yet, the advantage of being able to selectively control the relative sensory contributions to the final percept came at the price of not obtaining the multisensory benefit (i.e., a reduction in variance for the perceived signal location) that is afforded by reliability-weighted integration according to MLE principles (Ernst and Banks, 2002; Alais and Burr, 2004).

Next, we investigated how auditory and visual signals were integrated into spatial representations at the neural level, focusing on low-level visual areas (V1–3), low-level auditory areas (primary auditory areas and planum temporale), and parietal areas (IPS0–4). Combining fMRI multivariate decoding with classic MLE analysis (as in our psychophysics analysis), we obtained neural weights and variances of spatial representations from neurometric functions that were computed based on spatial locations decoded from regional BOLD-response patterns.

Consistent with previous reports of multisensory influences and interactions in primary sensory areas (Foxe et al., 2000; Bonath et al., 2007, 2014; Kayser et al., 2007; Lakatos et al., 2007; Lewis and Noppeney, 2010; Werner and Noppeney, 2010; Lee and Noppeney, 2014), unisensory auditory signals elicited spatial representations in visual cortex and visual signals in auditory cortex. In other words, unisensory signals from non-preferred sensory modalities can be decoded from low-level sensory areas (Meyer et al., 2010; Liang et al., 2013; Vetter et al., 2014). Yet, the unisensory neurometric functions demonstrated that the spatial representation decoded from low-level visual and auditory areas were far more reliable for signals from the preferred than nonpreferred sensory modality. As a result and in line with MLE predictions, the sensory weights applied during multisensory integration to the signals from the auditory modality were negligibly small in low-level visual areas. In auditory areas, the visual weight was also small, at least during auditory report, but significantly different from 0. Further, neither the sensory weights nor the variance depended significantly on the reported sensory modality. Instead the variance of the spatial representation decoded from audiovisual signals was comparable to the unisensory visual variance in visual regions and comparable to unisensory auditory variance in auditory regions. Hence, our quantitative analysis based on neurometric functions moves significantly beyond previous research that demonstrated better-than-chance decoding performance for auditory signals from visual areas and vice versa (Meyer et al., 2010; Liang et al., 2013; Vetter et al., 2014). It demonstrates that signals from the nonpreferred sensory modality elicit representations that are far less reliable than those evoked by signals from the preferred sensory modality. Likewise, nonpreferred signals exert only limited influences on spatial representations in low-level sensory areas during audiovisual stimulation. Surprisingly, visual signals exerted stronger influences on auditory areas than vice versa, potentially reflecting the importance of visual inputs for spatial perception (Welch and Warren, 1980).

In higher-order areas IPS0–4, unisensory auditory and visual signals elicited spatial representations that were more comparable in their reliabilities. Yet, consistent with the well-known visual response properties of IPS0–4 (Swisher et al., 2007; Wandell et al., 2007), visual stimulation elicited more reliable representations. Hence, as predicted by MLE, IPS0–4 gave a stronger weight to the visual signal during multisensory integration. Potentially, IPS could implement reliability-weighted integration via probabilistic population codes (Ma et al., 2006) or normalization over the pool of neurons within a region (Ohshiro et al., 2011, 2017). Because we used a linear SVM classifier as decoder, it remains unclear which encoding scheme IPS used to represent audiovisual space. To investigate the potential neural implementations, future studies may use explicit encoding models (e.g., estimating voxels’ tuning function for space using population receptive fields methods; Dumoulin and Wandell, 2008) to characterize the effects of reliability-weighted multisensory integration on voxel-response tuning functions.

However, in contrast to the MLE predictions, the sensory weights in IPS were modulated not only by visual reliability, but also by the sensory modality that needed to be reported. The visual signal had a stronger influence on the decoded spatial representation during visual than auditory report, thereby reflecting the sensory weight profile observed at the behavioral level. Likewise, the variance of the spatial representation for audiovisual stimuli in IPS0–4 was marginally influenced by the modality of the reported signal, suggesting that the formation of audiovisual representations in IPS0–4 may be susceptible to top-down control. Dynamic causal modeling and Bayesian model comparison suggested that these changes in audiovisual spatial representations in IPS0–4 were mediated by modulatory effects: visual reliability modulated the bottom-up connections from V1–3 to IPS0–4, and modality-specific report modulated the top-down connections from PFC to IPS0–4.

Our results demonstrate that observers do not fully integrate auditory and visual signals into unified spatial representations at the behavioral level and the neural level in higher-order association areas IPS0–4. Even when auditory and visual signals were spatiotemporally congruent and hence likely to originate from a common source, the sensory signal that needed to be reported had a stronger influence on the spatial representations than the one that was to be ignored. An important aim for future studies is to determine how a change in reported sensory modality modulates audiovisual integration and to dissociate between two main mechanisms: First, modality-specific report may influence the sensory weights via attentional mechanisms. Attention is known to increase the signal-to-noise ratio or reliability of the signal in the attended sensory modality (Desimone and Duncan, 1995; Martinez-Trujillo and Treue, 2004; Briggs et al., 2013; Sprague et al., 2015). Thereby, attention mediates a greater weight in the multisensory integration process (Alsius et al., 2005, 2007; Busse et al., 2005; Talsma and Woldorff, 2005; Talsma et al., 2007, 2010; Zimmer et al., 2010a, b; Donohue et al., 2011; Vercillo and Gori, 2015; Macaluso et al., 2016; but see Helbig and Ernst, 2008). In this model, auditory and visual signals are integrated weighted by their sensory reliabilities. Yet, in contrast to the MLE model, the reliability of each sensory input can be modified before audiovisual integration by top-down attention as manipulated by modality-specific report. Second, modality-specific report instructs participants not to fuse signals into one unified percept but to form a spatial estimate selectively for one of the two signals. These instructions may attenuate the integration process even for signals that are collocated in space, thereby enabling participants to compute a final spatial estimate that is more strongly based on the reported sensory modality. In this second case, MLE analyses compute a stronger weight for the reported signal because of its task relevance rather than attentionally increased sensory reliability. Yet, human behavior in this second case is better accommodated by recent Bayesian causal inference models that explicitly model the potential causal structures of the multisensory signals—that is, whether they have been caused by common or independent causes (Körding et al., 2007; Shams and Beierholm, 2010; Wozny et al., 2010; Rohe and Noppeney, 2015a, 2016). In Bayesian causal inference, a final estimate of the spatial location under auditory or visual report is obtained by combining the estimates under the two causal structure, i.e., the MLE reliability-weighted estimate under the assumption of a common source and the estimate of the sensory signals that needs to be reported under the assumption of independent causes. Because the underlying causal structure is uncertain and modality-specific report instructions may further lower the observers’ belief that signals are caused by a common source, the reported spatial estimates differ for auditory and visual reports, thereby modeling effects of modality-specific report. Further, because in the course of our experiment the audiovisual signals were spatially uncorrelated across all conditions (i.e., the auditory and the visual signal locations were independently sampled from the four locations; Fig. 1*B*), participants might have implicitly learned a low prior probability of a common cause. Thus, even in conditions in which the audiovisual signals had only a small spatial disparity (which we selectively used in our analyses), participants might have computed a low posterior belief that signals arose from a common cause. In general, previous research has shown that Bayesian causal inference outperforms the MLE model under conditions in which a common cause is unlikely, for example a large spatial discrepancy between the audiovisual signals (Körding et al., 2007; Rohe and Noppeney, 2015a, b). To dissociate the effects of modality-specific attention and report, future studies may use attentional cuing paradigms that pre-cue participants before stimulus presentation to attend to the visual (respective to auditory) signal and post-cue them after stimulus presentation to report the location of the auditory (respective to visual) signal.

To conclude, the present study characterized how the brain integrates auditory and visual signals into spatial representations and how these integration processes are modulated by modality-specific report or attention. Combining psychophysics and multivariate fMRI decoding, we demonstrated that classic MLE models cannot fully account for participants’ behavioral and neural responses if the experimental context (i.e., modality-specific report and overall uncorrelated audiovisual signals) undermines observers’ perception of a common signal cause, thus violating the MLE model’s core assumption. Although the behavioral and neural weights in parietal cortex depended on the relative sensory reliabilities in line with the quantitative predictions of the MLE model, they were also modulated by whether participants attended and reported the visual or the auditory signal location. Likewise, the variance of the spatial representations depended on task context to some extent, even for collocated audiovisual signals, at both neural and behavioral levels. These results suggest that audiovisual integration can be modulated by top-down control. Even when the auditory and visual signals were spatially close (or collocated) and temporally synchronous, modality-specific report influenced how they were weighted and integrated into spatial representations.

## Synthesis

Reviewing Editor: Lila Davachi, New York University Decisions are customarily a result of the Reviewing Editor and the peer reviewers coming together and discussing their recommendations until a consensus is reached. When revisions are invited, a fact-based synthesis statement explaining their decision and outlining what is needed to prepare a revision will be listed below. The following reviewer(s) agreed to reveal their identity: Michael Landy, Marko Nardini.

Both reviewers found that the paper still needed work to better situate the current experiment and findings into the current literature. Specifically, because the task used here does not require that participants attend to both cues and that the statistical regularities of the task may decouple the cues, both reviewers felt this task and results were interesting but may be one possibility for how cue integration works. So, to deal with this, they ask that the actual result should be “ more clearly interpreted as something that CAN happen, rather than is a necessary part of audiovisual integration”. In the title, end of conclusion, and significance statement, “IS modulated by.. / IS flexibly adjusted by” would be better as “CAN BE modulated / adjusted by..”. Along the same lines, both reviewers were concerned about the way ‘optimal’ was introduced and how the prior literature was cited. As noted above, the findings may have come about because of task relevance (as noted in the paper), but may also be due in part to experiencing highly uncorrelated statistics of the two kinds of stimuli throughout the experiment.

On a related note, Rev 2 writes “The notion of ‘mandatory integration’ isn't assumed in all cue integration papers (despite, e.g, lines 222-223). In fact, the Hillis et al. paper (Science), of which I'm a co-author and which is cited here, suggests that integration is mandatory for within-modality integration, but isn't for cross-modal integration (in their case: vision and haptics).” This reviewer also notes “ The task here effectively asks the participant not to integrate, but rather to report a single modality. Nevertheless, this is also a situation that has been studied before, and modeled using ideas of causal inference and model selection or model averaging. The main/first paper on that is Körding's, analyzing data for the same cross-modal integration problem studied here, and yet that doesn't show up until the Discussion. That paper shows that humans, in some situations, can't ignore the other cue even when asked to do so. In fact, they provide a generative model that suggests one shouldn't ignore the cue. This paper is based entirely on that situation, and yet a model for what is meant by ‘optimal’ in this case never shows up in the Introduction. To me, that's really problematic.” Please address these issues in your revision.

There was also a concern about now presenting a forward hypothesis for what brain regions should be representing or coding. Rev 2 asks “First, why should different brain areas be confronted with different cue reliabilities and thus do independent versions of optimal cue integration compared to other brain areas?” No theory is stated. Please address how the linear classification could be improved in future work. I am including Reviewer 2's comments here as a seed for thought on your part. You do not need to address the issue with data but perhaps mention it in the Discssion. Rev 2 writes “I prefer fMRI analysis based on a forward model of the underlying computation, rather than just throwing a linear classifier at the voxels. But here, you really should have a decent forward model, at least in the visual areas: the population receptive fields of the individual neurons. That is, you could have used a PRF estimate from a retinotopy scan and actually computed an estimate of stimulus location based on it on each trial, and used that value for the forced-choice task. That would make much more sense to me. ”

Minor points:

* Line 47: Are they really unexplored? I thought exploring optimal cue integration in the brain was exactly what Ban et al. (cited 3 lines above) set out to do. 

* Lines 142-143: I was confused by these lines, because the whole setup was about left-right discrimination, and now we are told it is 4AFC. Also ‘signal locations’ in this sentence is slightly unclear. It would be better to spell out the 4 choices, and state here that in the current paper you are treating it as 2AFC (I can't remember when you state that, but it's a bit later). When I read this line, I wondered why the early figures had psychometric functions even though this was 4AFC. Better to give the reader what s/he needs when s/he needs to know it. You don't state this until line 205. 

* 151: What's a ‘null-event’, and why are they included?

* 192: This equation really isn't intelligible without defining the terms. It's correct, but only because your PSE terms are defined (well, should have been explicitly defined) based on the x-axis you use in your plots (based on the average of the two cues' values when there is a perturbation).

* 227-229: First, I have no idea what the text “left and right hemi-field” is doing here. Second, I can't figure out how you got to 17 parameters. I got: 1 lapse, 2x3 for slope and PSE for unimodal, 2 more PSEs and 1 slope for 2x2 AV PSEs for bimodal, which yields 19 parameters. As a result, I couldn't match any later parameter counts either (e.g., line 239's count of 21). 

* 310: I'm not a big fan of blurring fMRI data spatially. Why was that done here? It just loses information that is potentially useful for later analyses (such as MVPA). 

* 326-327: I think you should spell out exactly what ‘within each condition’ means here. 

* Line 370: “Absolute values were used to compute a two-tailed test”. Do you mean you did a one-tailed test on absolute values, thus effectively doing a two-tailed test? If so, say so, and say why you did it this way. Seemed opaque to me. 

* Line 381 et seq.: I'm not sure what this version of a bootstrapped null distribution is (by looking at residuals and calling them a null distribution). Why not derive it in the usual way, by permuting the labels on the raw data and re-fitting? 

* 533: ‘classicial’ 

* 582: It's odd to refer to the ‘audiovisual’ percept when observers were explicitly instructed NOT to report an audiovisual percept, but rather a single modality. 

* 584-585: I didn't check, but again this paper is a bit about whether there is conscious access to uni-modal signals unperturbed by the other modality. Do you check for significant different in the weights for the reported modality different from one? 

* Figure 4: What are the unnormalized sigma values? I don't see that you ever provide them. In particular, the auditory values are only about 5x the visual ones, and hence are actually, I'd imagine, pretty good. And, of course, the auditory signals in visual areas are presumably very weak, thus low S/N, and that will blow up the sigma values, but might not reflect the information available in the local neurons. 

* 760: “remains controversially discussed” doesn't parse 
