# Supplementary figures and images for: Reliability-Weighted Integration of Audiovisual Signals Can Be Modulated by Top-down Attention
Source: eNeuro. 2018 Mar 8;5(1):ENEURO.0315-17.2018. doi: 10.1523/ENEURO.0315-17.2018 (PMC5844059; doi:10.1523/ENEURO.0315-17.2018)

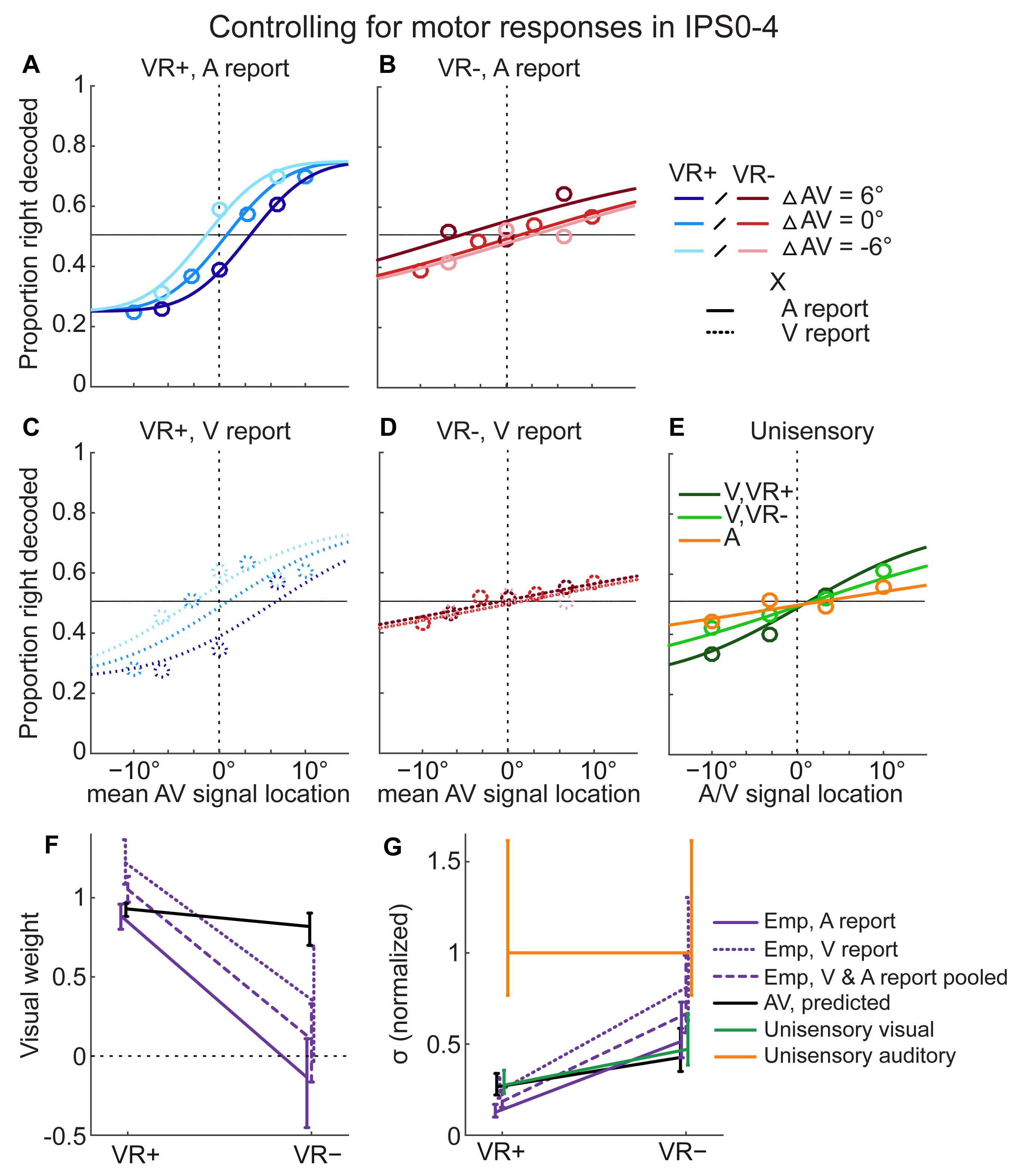

Supplement: Figure 3-1 — fMRI results in the intraparietal sulcus when controlling for motor responses: neurometric functions, visual weights and audiovisual variances. In intraparietal sulcus (IPS0–4), neurometric functions were fitted to the fraction of decoded “right” location responses plotted as a function of the mean audiovisual (AV) location (see figure 2 legend for additional information). To control for motor planning in IPS0–4, activation patterns were obtained from a general linear model that modeled participants’ trial-wise button responses as a nuisance variable. A–D, Neurometric functions are plotted separately for the four conditions in our 2 (visual reliability: high, VR+ vs. low, VR-) x 2 (modality-specific report: auditory vs. visual) factorial design. E, In unisensory conditions, psychometric functions were fitted to the fraction of right location responses plotted as a function of the signal location from unisensory auditory (A) and visual conditions of high (V, VR+) and low (V, VR-) visual reliability. F, Visual weights (mean and 68% bootstrapped confidence interval): MLE predicted and empirical visual weights for 2 (visual reliability: high, VR+ vs. low, VR-) x 2 (modality-specific report: auditory vs. visual) AV conditions. To facilitate the comparison with the MLE predictions that do not depend on modality-specific report, the visual weights are also plotted after pooling the data across both report conditions and re-fitting the neurometric functions. G, Standard deviations (σ, mean and 68% bootstrapped confidence interval): Unisensory and audiovisual MLE predicted and empirical standard deviations for the same combination of conditions as in F. For illustrational purposes standard deviations were normalized by the auditory standard deviation. Download Figure 3-1, TIF file. [file sup_enu-eN-NWR-0315-17-s02.tif]

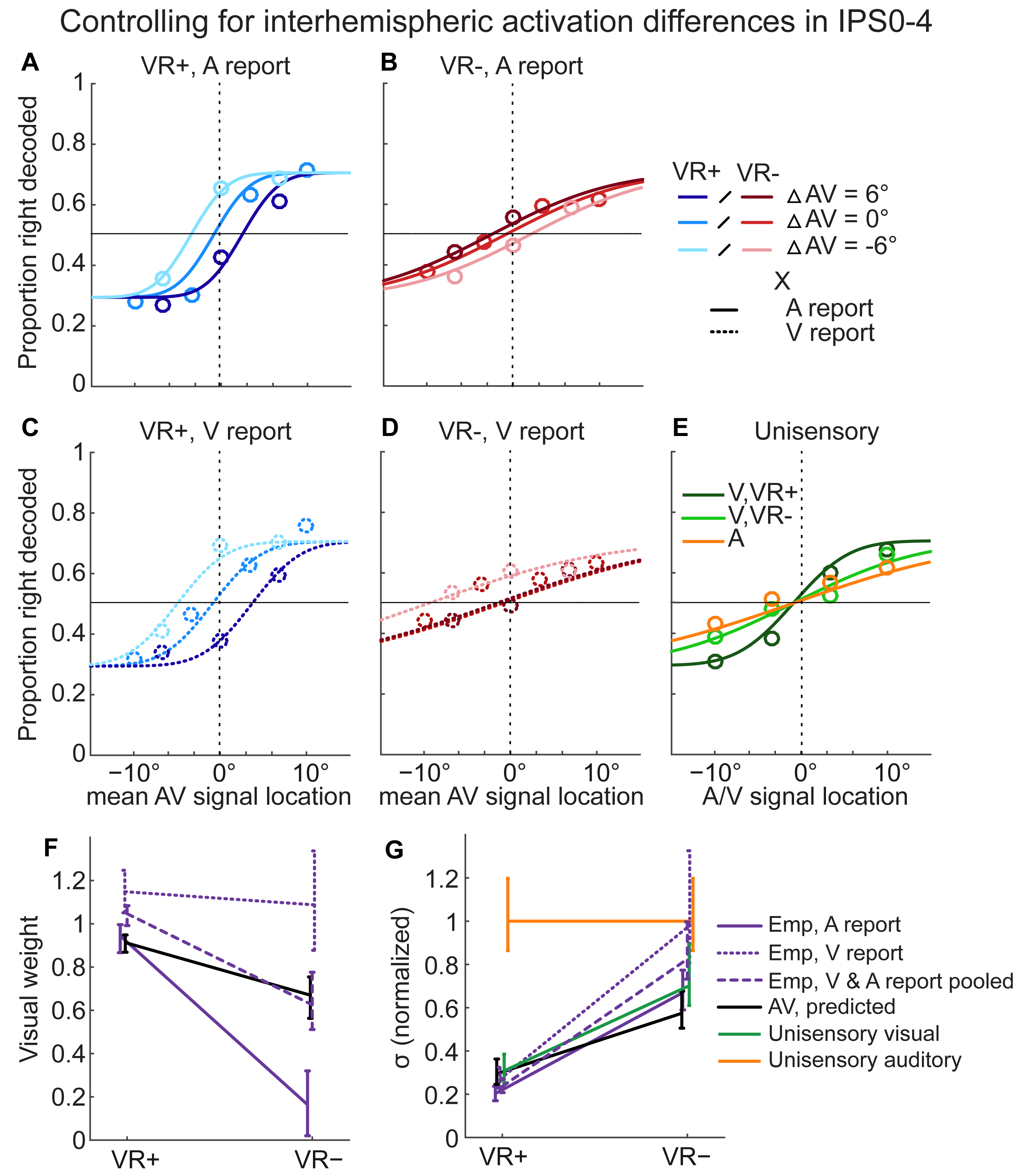

Supplement: Figure 3-2 — fMRI results in the intraparietal sulcus when controlling for global interhemispheric activation differences: neurometric functions, visual weights and audiovisual variances. In intraparietal sulcus (IPS0–4), neurometric functions were fitted to the fraction of decoded “right” location responses plotted as a function of the mean audiovisual (AV) location (see figure 2 legend for additional information). To control for global interhemispheric activation differences, activation patterns were z normalized separately for the left and right hemisphere within each condition prior to multivariate decoding. A–D, Neurometric functions are plotted separately for the four conditions in our 2 (visual reliability: high, VR+ vs. low, VR-) x 2 (modality-specific report: auditory vs. visual) factorial design. E, In unisensory conditions, psychometric functions were fitted to the fraction of right location responses plotted as a function of the signal location from unisensory auditory (A) and visual conditions of high (V, VR+) and low (V, VR-) visual reliability. F, Visual weights (mean and 68% bootstrapped confidence interval): MLE predicted and empirical visual weights for 2 (visual reliability: high, VR+ vs. low, VR-) x 2 (modality-specific report: auditory vs. visual) AV conditions. To facilitate the comparison with the MLE predictions that do not depend on modality-specific report, the visual weights are also plotted after pooling the data across both report conditions and re-fitting the neurometric functions. G, Standard deviations (σ, mean and 68% bootstrapped confidence interval): Unisensory and audiovisual MLE predicted and empirical standard deviations for the same combination of conditions as in F. For illustrational purposes standard deviations were normalized by the auditory standard deviation. Download Figure 3-2, TIF file. [file sup_enu-eN-NWR-0315-17-s03.tif]

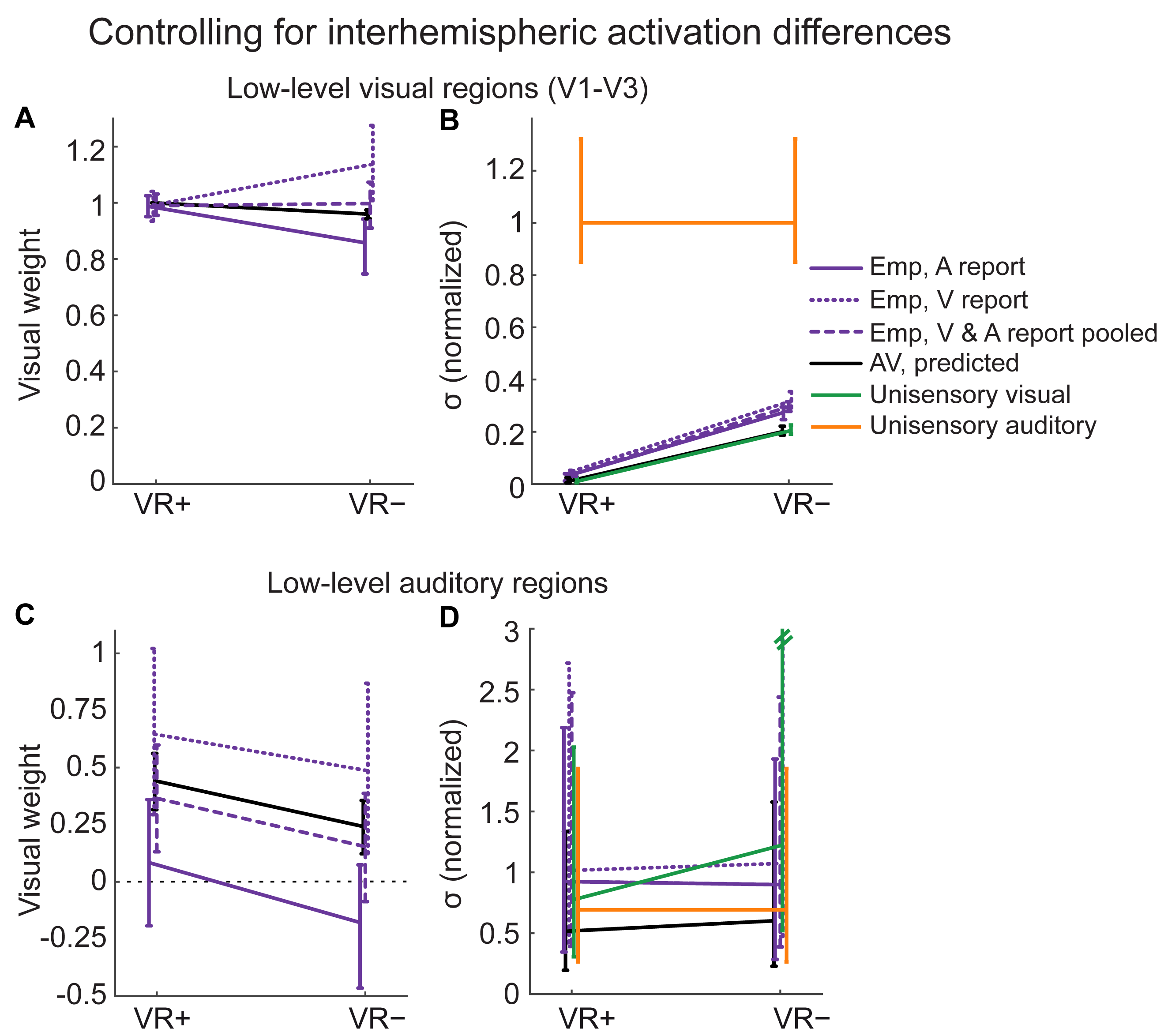

Supplement: Figure 4-1 — fMRI results in low-level visual and auditory regions when controlling for interhemispheric activation differences: Visual weights and audiovisual variances. To control for interhemispheric activation differences, activation patterns were z normalized separately in the left and right hemisphere within each condition prior to multivariate pattern decoding. A, Visual weights (mean and 68% bootstrapped confidence interval): MLE predicted and empirical visual weights for 2 (visual reliability: high, VR+ vs. low, VR-) x 2 (modality-specific report: auditory vs. visual) audiovisual conditions in low-level visual regions (V1-3). To facilitate the comparison with the MLE predictions that do not depend on modality-specific report, the visual weights are also plotted after pooling the data across both report conditions and re-fitting the neurometric functions. B, Standard deviations (σ, mean and 68% bootstrapped confidence interval): Unisensory and audiovisual MLE predicted and empirical standard deviations for the same combination of conditions as in A. For illustrational purposes standard, deviations were normalized by the auditory standard deviation. C, Visual weights (mean and 68% bootstrapped confidence interval): MLE predicted and empirical visual weights in low-level auditory regions (hA) as shown in A. D, Standard deviations (σ, mean and 68% bootstrapped confidence interval): Unisensory and audiovisual MLE predicted and empirical standard deviations of spatial representations in low-level auditory regions (hA) as shown in B; note that the upper confidence interval for the visual variance is truncated for illustrational purposes. For illustrational purposes, standard deviations were normalized by a combined visual standard deviation for low and high visual reliability. Download Figure 4-1, TIF file. [file sup_enu-eN-NWR-0315-17-s04.tif]
